# Glucagon-like peptide-1 receptor expression after myocardial infarction: Imaging study using ^68^Ga-NODAGA-exendin-4 positron emission tomography

**DOI:** 10.1007/s12350-018-01547-1

**Published:** 2018-12-13

**Authors:** Mia Ståhle, Ville Kytö, Max Kiugel, Heidi Liljenbäck, Olli Metsälä, Meeri Käkelä, Xiang-Guo Li, Vesa Oikonen, Pekka Saukko, Pirjo Nuutila, Juhani Knuuti, Anne Roivainen, Antti Saraste

**Affiliations:** 1 Turku PET Centre, University of Turku, 20520 Turku, Finland; 2 Heart Center, Turku University Hospital, Turku, Finland; 3 Research Centre of Applied and Preventive Cardiovascular Medicine, University of Turku, Turku, Finland; 4 Turku Center for Disease Modeling, University of Turku, Turku, Finland; 5 Turku PET Centre, Åbo Akademi University, Turku, Finland; 6 Department of Pathology and Forensic Medicine, University of Turku, Turku, Finland; 7 Department of Endocrinology, Turku University Hospital, Turku, Finland; 8 Turku PET Centre, Turku University Hospital, Turku, Finland

**Keywords:** Glucagon-like peptide-1 receptor, myocardial infarction, inflammation, PET

## Abstract

**Background:**

Activation of glucagon-like peptide-1 receptor (GLP-1R) signaling protects against cardiac dysfunction and remodeling after myocardial infarction (MI). The aim of the study was to evaluate ^68^Ga-NODAGA-exendin-4 positron emission tomography (PET) for assessment of GLP-1R expression after MI in rats.

**Methods and Results:**

Rats were studied at 3 days, 1 and 12 weeks after permanent coronary ligation or a sham-operation. Rats were injected with ^68^Ga-NODAGA-exendin-4 and scanned with PET and contrast-enhanced computed tomography (CT) followed by digital autoradiography and histology of left ventricle tissue sections. ^68^Ga-NODAGA-exendin-4 PET/CT showed focally increased tracer uptake in the infarcted regions peaking at 3 days and continuing at 1 week after MI. Pre-treatment with an unlabeled exendin-4 peptide significantly reduced ^68^Ga-NODAGA-exendin-4 uptake. By autoradiography, ^68^Ga-NODAGA-exendin-4 uptake was 8.6-fold higher in the infarcted region and slightly increased also in the remote, non-infarcted myocardium at 1 week and 12 weeks post-MI compared with sham. Uptake of ^68^Ga-NODAGA-exendin-4 correlated with the amount of CD68-positive macrophages in the infarcted area and alpha-smooth muscle actin staining in the remote myocardium.

**Conclusions:**

^68^Ga-NODAGA-exendin-4 PET detects up-regulation of cardiac GLP-1R expression during healing of MI in rats and may provide information on the activated repair mechanisms after ischemic myocardial injury.

**Electronic supplementary material:**

The online version of this article (10.1007/s12350-018-01547-1) contains supplementary material, which is available to authorized users.

## Introduction

Glucagon-like peptide-1 (GLP-1) is an intestinal hormone stimulating insulin secretion in response to nutrients. In addition to pancreatic β-cells, glucagon-like peptide-1 receptor (GLP-1R) is expressed in many other tissues and has effects on cardiovascular function.^1^ Pharmacological activation of GLP-1R signaling reduces cardiovascular events in diabetic patients,^1^ and has been shown to protect from ischemic myocardial injury in experimental models^2^–^4^ and human myocardial infarction (MI).^5^–^7^ Cardioprotective effects of GLP-1 involve attenuation of myocardial inflammatory response^8^–^11^ and fibrosis^8^,^9^,^12^ after ischemic injury protecting against cardiac dysfunction and chamber dilatation after MI.^8^,^9^,^11^–^13^ However, cardiac GLP-1R expression during healing of MI and left ventricle (LV) remodeling remains uncertain.

Molecular imaging agents have been developed for specific detection of GLP-1R in insulin-secreting pancreatic β-cells and insulinomas.^14^,^15^ Recently, positron emission tomography (PET) with ^18^F-FBEM-Cys^40^-exendin-4, revealed up-regulation of GLP-1R expression in the myocardium after acute ischemic myocardial injury in rats.^16^ However, the relationship between GLP-1R expression and the mechanisms involved in healing of MI and LV remodeling remains unknown. We have previously validated another tracer, ^68^Ga-NODAGA-exendin-4 for PET imaging of GLP-1R on pancreatic β-cells in healthy rats.^17^^68^Ga-NODAGA-exendin-4 can be produced with high specific radioactivity, is relatively stable in vivo, has high binding affinity to GLP-1R, and is rapidly cleared from the blood.^17^

In the present study, we investigated whether ^68^Ga-NODAGA-exendin-4 PET/computed tomography (CT) imaging can detect up-regulation of GLP-1R expression in the rat heart after MI and whether tracer uptake is associated with histological markers of myocardial repair. Myocardial uptake of ^68^Ga-NODAGA-exendin-4 was studied by a small-animal PET/CT camera and autoradiography of LV tissue sections at 3 days, 1 or 12 weeks after the MI. Specificity of tracer uptake was studied by injection of unlabeled exendin-4 peptide prior to ^68^Ga-NODAGA-exendin-4.

## Methods

### Animal Model and Study Design

The national Animal Experiment Board in Finland and the Regional State Administrative Agency for Southern Finland approved the studies. They were carried out in compliance with the European Union directive relating to the conduct of animal experimentation. In total, 80 male Sprague-Dawley rats (Central Animal Laboratory, University of Turku, Turku, Finland) weighing 283 ± 29 g were utilized. Myocardial infarction was induced by a permanent ligation of the left coronary artery (LCA) using the previously described method.^18^ The sham-operation consisted of the same protocol except for the ligation of the LCA.^18^ See Supplemental Figure 1 for a flow diagram explaining experiments.

The final study group for evaluation of myocardial uptake of ^68^Ga-NODAGA-exendin-4 consisted of 22 rats with coronary ligation and MI studied at 3 days, 1 or 12 weeks, and 18 sham-operated rats studied at 1 or 12 weeks (Table 1). In addition to this, 3 rats with MI were used for competition experiments and three rats with MI for ex vivo biodistribution analysis 1 week after coronary occlusion.

**Table 1:** Characteristics of study animals

	3 Days	1 Week		12 Weeks	
	MI (n=6)	Sham (n=9)	MI (n=7)	Sham (n=9)	MI (n=9)
Blood glucose (mmol/L)	7.3 ± 1.5	9.9 ± 2.0	6.6 ± 0.94*	9.0 ± 2.3	10 ± 2.3
Body weight (g)	270 ± 9.6	290 ± 25	290 ± 40	450 ± 29	480 ± 35
LV mass (g)	0.74 ± 0.080	0.78 ± 0.040	0.92 ± 0.16*	1.01 ± 0.028	1.2 ± 0.13*
LV mass/body weight	0.0028 ± 0.00024	0.0027 ± 0.00020	0.0032 ± 0.00041*	0.0022 ± 0.00013	0.0025 ± 0.00037*
MI size (%)	48 ± 7.4		42 ± 6.7		35 ± 14

Values are mean ± SD

*LV*, left ventricle; *MI*, myocardial infarction

*P < 0.01 MI vs Sham

During all imaging studies, rats were anesthetized with isoflurane (4-5% for induction and 1.5-2% for maintenance) and body temperature was maintained using a heating pad.

### Echocardiography

The method is described in the Supplementary data.

### Radiochemistry

The structure of [Nle^14^,Lys^40^(Ahx-NODAGA)NH_2_]-exendin-4 peptide is presented in Supplemental Figure 2. ^68^Ga-NODAGA-exendin-4 was prepared as described previously.^17^ In vivo stability and radiometabolism of ^68^Ga-NODAGA-exendin-4 in plasma and urine were studied by radio-high-performance liquid chromatography^17^ 12 weeks after the sham-operation (n= 3).

### PET/CT

Protocols for PET/CT and image analysis have been described earlier.^18^ In brief, after fasting for 4 hours, 1.4 ± 0.15 mCi of ^68^Ga-NODAGA-exendin-4 (52 ± 5.5 MBq, 3.4 ± 1.6 nmol, 16 ± 7.8 µg, 250-700 µL) was injected intravenously via the tail vein and the animals were scanned using a small-animal PET/CT (Inveon Multimodality, Siemens Medical Solutions, Knoxville, TN, USA) for 60 minutes starting from the time of the injection. Immediately after the PET, 250 µL of intravascular iodinated contrast agent (eXIATM160XL, Binitio Biomedical Inc., Ottawa, ON, Canada) was injected and a contrast-enhanced CT was acquired.

Quantitative PET analyses were performed using Carimas v.2.8 software (Turku PET Centre, Turku, Finland) using heart tools. PET and CT images were automatically superimposed. The regions of interest (ROIs) were drawn corresponding to the infarcted region in the myocardium supplied by the LCA and remote, non-infarcted myocardium in the inferior septum. Endocardial border was delineated and myocardium localized on high resolution contrast-enhanced CT. In sham-operated rats, ROIs were drawn in the anterolateral wall corresponding LCA territory. The organs adjacent to the heart; chest wound, liver, lungs and kidneys were also analyzed. Optimal contrast between ^68^Ga-NODAGA-exendin-4 uptake in the infarcted area and blood pool was observed in the last three time frames, and therefore regional standardized uptake values (SUV_mean_) were calculated from the mean radioactivity concentrations (Bq/mL) 50-60 minutes after injection. Coefficient of variation for repeated measurements of tracer uptake in the infarcted area was 14%.

### Kinetic Modeling

Since ^68^Ga-NODAGA-exendin-4 has been shown to internalize into cells,^19^ graphical Patlak analysis^20^ was used to estimate irreversible tracer uptake as net influx rate (*K*_i_). Metabolite-corrected plasma time-activity curves were used as input function in Patlak analysis. Image-derived blood curves measured in the LV cavity were converted into metabolite-corrected plasma curves using the group median plasma-to-blood ratio and percent of intact ^68^Ga-NODAGA-exendin-4 measured in the metabolite analysis (Supplemental Figure 3). *K*_i_ was calculated as the slope of the plot after the linear phase was reached 20 minutes after tracer injection. Parametric images of the net influx rate were obtained by using Patlak plots, but the reported *K*_i_ values are based on analysis of regional time-activity curves.

### Biodistribution Analysis, Autoradiography and Histology

After the PET/CT imaging, at 80 minutes post-injection of ^68^Ga-NODAGA-exendin-4, blood was collected by cardiac puncture and rats were sacrificed by cervical dislocation. The heart was rinsed with saline to remove excess blood and the LV was excised. For biodistribution analysis, samples from MI region, remote myocardium, blood, pancreas and spleen were prepared, and total radioactivity was measured using a gamma counter (Triathler 3”, Hidex Oy, Turku, Finland). Radioactivity values were normalized for injected radioactivity dose per animal weight, decay, and the weight of the tissue sample. Results were expressed as SUV.

For autoradiography, the excised LV was frozen in cooled isopentane and cut into serial 20 µm transverse cryosections at 1 mm intervals from the apex to base. Autoradiography was performed using the previously described method.^18^ Then, sections were stained with hematoxylin and eosin, scanned with a digital slide scanner (Pannoramic 250 Flash, 3DHistech Ltd., Budapest, Hungary) and superimposed with autoradiographs.

Histology was studied in serial LV cryosections or paraffin embedded sections. Masson’s trichrome (Sigma-Aldrich, St. Louis, MO, USA) stained 8 µm cryosections were used to determine the MI size and measure collagen density. To compare ^68^Ga-NODAGA-exendin-4 uptake with histological markers of myocardial repair, 8 µm LV cryosections were stained with a macrophage CD68 antibody or alpha-smooth muscle actin (α-SMA) antibody. In order to study the localization of GLP-1R, additional samples of the LV (n= 4 MI and n= 2 Sham) were fixed overnight in formalin and embedded in paraffin. Serial 4 μm paraffin sections were stained with a GLP-1R antibody. For studying co-localization with macrophages and α-SMA expressing interstitial cells, paraffin sections were double immunofluorescence stained with GLP-1R and either a CD68 or α-SMA antibody. (Supplemental Table 1) The GLP-1R staining protocol was optimized using pancreatic sections as a positive control and healthy myocardium that did not show staining as a negative control. Analysis of autoradiographs and histology is described in the Supplementary data.

### Competition Experiment

To assess the specificity of ^68^Ga-NODAGA-exendin-4 accumulation, 12 mg/kg of unlabeled exendin-4 peptide (ChinaPeptides Co. Ltd., Shanghai, China) was injected 10 minutes before ^68^Ga-NODAGA-exendin-4 injection in order to block specific binding sites 1 week after coronary ligation. The rats underwent PET/CT imaging followed by autoradiography study.

### Statistical Analysis

The results are presented as mean ± standard deviation (SD). Data were analyzed using SPSS Statistics software v. 22 (IBM, NY, USA). Normality was examined by a Shapiro-Wilk test, and equality of variances was tested with Levene’s test. For comparisons between the two groups, a Student *t*-test for unpaired or paired data was used. Multiple comparisons were done by one-way analysis of variance (ANOVA) followed by a Tukey–Kramer correction. A Spearman correlation was used to analyze correlation between two continuous variables. A *P* value of less than 0.05 was considered statistically significant.

## Results

Basic characteristics of animals are presented in Table 1. None of the sham-operated rats showed myocardial injury, whereas there was a transmural MI in all 22 rats with coronary ligation included in the final study group. The average size of the MI was medium-to-large and it was comparable between day 3, week 1 and week 12 time-points. The LV mass normalized by body weight was higher at 1 (*P *= 0.010) and 12 weeks (*P *= 0.031) after MI compared with sham-operation. Echocardiography (Supplemental Table 2) showed significant LV enlargement from 1 to 12 weeks.

### Histology

**Figure 1:**
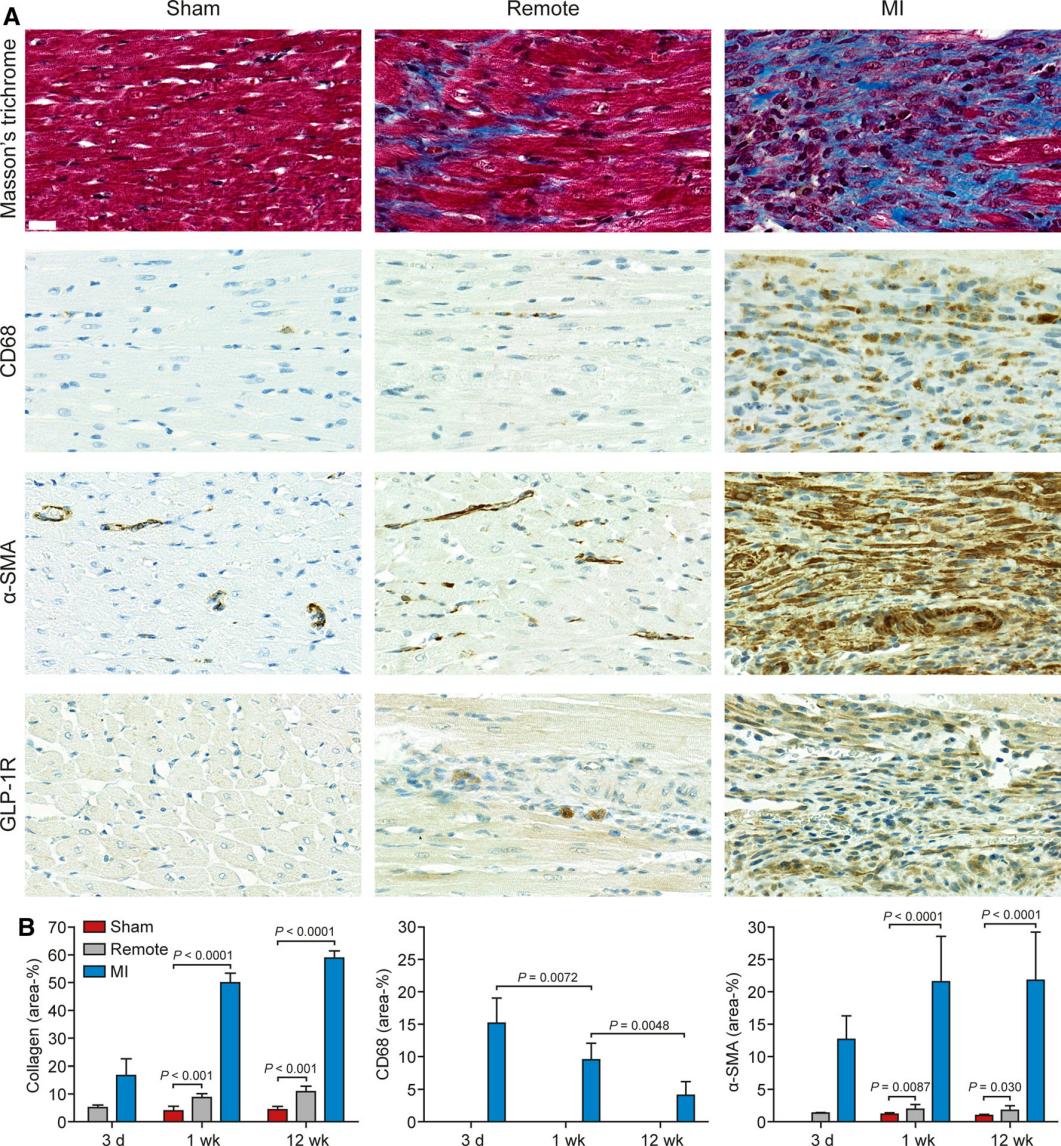
Histology of rat myocardium 1 week after coronary ligation or sham-operation. **A** Masson’s trichrome staining showing myocytes as red color in the left ventricle myocardium of a sham-operated rat, whereas collagen fibers (blue) are detected in the infarcted area and in the remote, non-infarcted myocardium. There are numerous CD68-positive macrophages, α-SMA-positive and GLP-1R-positive cells in the infarcted area, and scattered α-SMA- and GLP-1R-positive interstitial cells in the remote myocardium (brown color). Scale bar 20 μm. **B** Bars show the areas of collagen (Masson’s trichrome staining), CD68-positive staining and α-SMA-positive staining. Values are mean ± SD; Student’s *t*-test for unpaired measurements and ANOVA for comparisons of MI 3 day, 1 week and 12 week; n = 6-9 per each bar. (*α-SMA*, alpha-smooth muscle actin; *d*, day; *GLP-1R*, glucagon-like peptide-1 receptor; *MI*, myocardial infarction; *wk*, week)

Representative histological findings of rats are presented in Figure 1. Based on Masson’s trichrome staining, collagenous scar developed in the infarcted region. The area of interstitial fibrosis was small in the healthy myocardium of sham-operated rats (3.9 ± 1.6% at week 1 and 4.3 ± 1.2% at week 12), but increased in the remote myocardium after MI (8.7 ± 1.4% at week 1, *P* < 0.001 vs sham and 11 ± 2.0% at 12 weeks, *P* < 0.001 vs sham) (Figure 1).

There were very few CD68-positive macrophages in the myocardium of sham-operated rats and remote myocardium of rats with MI (areal percentage < 1%), but they were present in the infarcted area (Figure 1A), where the area of CD68 staining decreased gradually from day 3 (15 ± 3.9%) to week 1 (9.5 ± 2.6%, *P* = 0.0072) and week 12 (4.3 ± 1.2%, *P* = 0.0048) (Figure 1B).

There was intense α-SMA-positive staining in the infarcted area and scattered staining in the remote myocardium after MI (Figure 1A). The area of α-SMA staining was small in the myocardium of sham-operated rats (1.2 ± 0.23% at week 1 and 0.96 ± 0.18% at week 12) (Figure 1B). In the infarcted area, the area of α-SMA-positive staining was high and remained stable from 1 week (22 ± 7.0%, *P* < 0.0001 vs sham) until week 12 (22 ± 7.4%, *P* < 0.0001 vs sham) (Figure 1B). Compared with the sham-operation, the area of α-SMA staining also increased in the remote myocardium at 1 week (1.9 ± 0.73%, *P* = 0.0087) and at 12 weeks (1.8 ± 0.70%, *P* = 0.030) after MI (Figure 1B).

### Radiochemistry and In Vivo Stability of ^68^Ga-NODAGA-exendin-4

The specific radioactivity and radiochemical purity of ^68^Ga-NODAGA-exendin-4 were 35 ± 7 MBq/nmol and > 95% at the end of synthesis, respectively. According to the radio-high-performance liquid chromatography analysis in healthy, sham-operated rats at 80 minutes post-injection, > 63% of plasma radioactivity originated from the intact tracer and two radio-metabolites of ^68^Ga-NODAGA-exendin-4 were detected (Supplemental Figure 3).

### ^68^Ga-NODAGA-exendin-4 PET/CT

**Figure 2:**
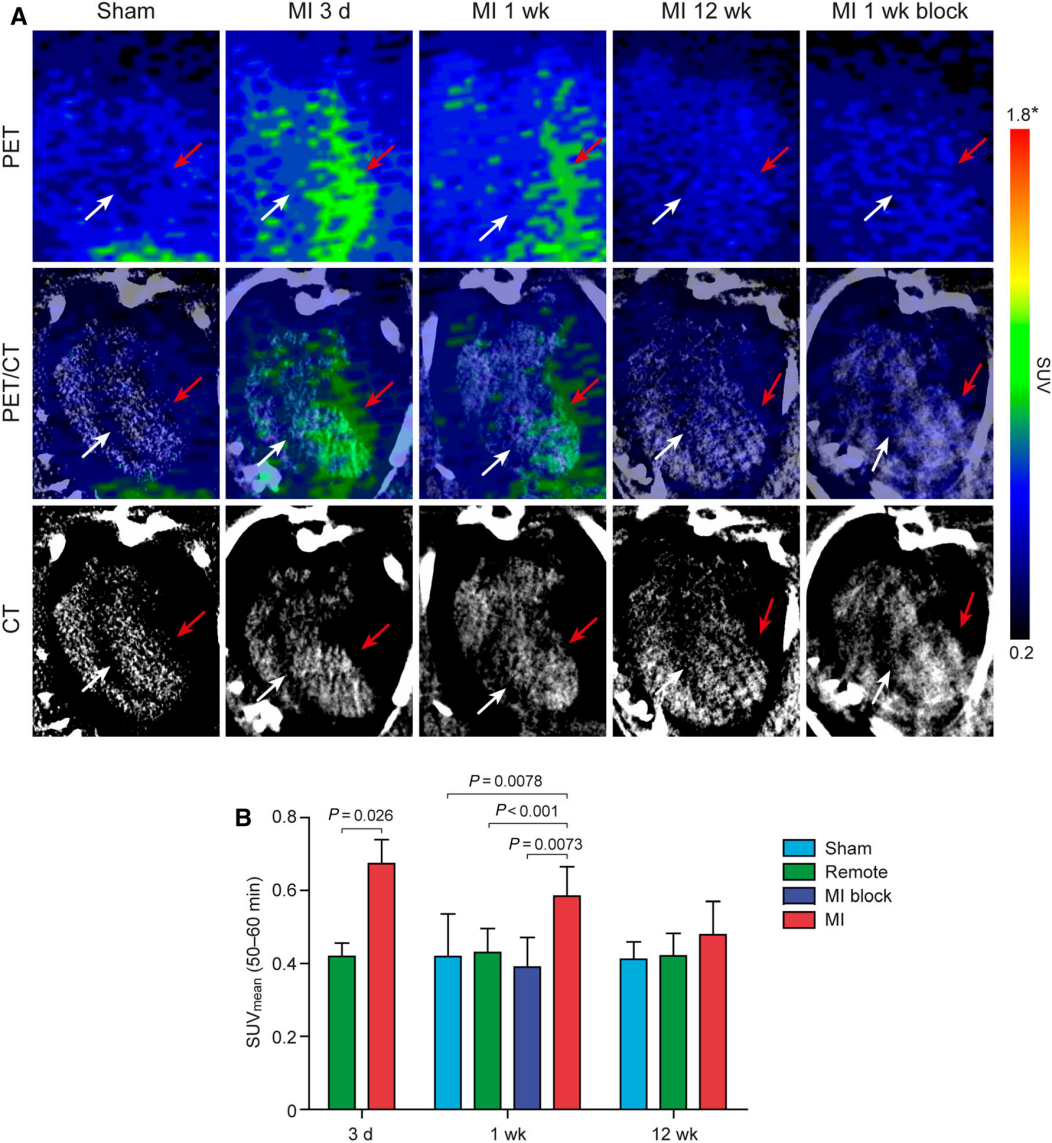
In vivo imaging of ^68^Ga-NODAGA-exendin-4 uptake. **A** Horizontal long axis positron emission tomography (PET) and corresponding contrast-enhanced computed tomography (CT) images showing focally increased ^68^Ga-NODAGA-exendin-4 uptake in the anterior wall of the left ventricle at 3 days and 1 week after myocardial infarction (MI) (red arrows) compared with remote myocardium (white arrows). After pre-injection of unlabeled exendin-4 peptide (MI 1 week block), uptake is as low as in the sham-operated rats. Scale; SUV_mean_ (50-60 minutes), *scale from 0.3 to 3.7 at 12 weeks. **B** Bars show the mean ± SD standardized uptake values (SUV_mean_) 50 to 60 minutes after injection. Student’s *t*-test for unpaired and paired (MI vs remote) measurements; 3 d n = 3, Sham 1 wk n = 8, MI 1 wk n = 7, MI block n = 3, Sham 12 wk n = 6, MI 12 wk n = 8. (*d*, day; *wk*, week)

There was no visible uptake of ^68^Ga-NODAGA-exendin-4 in the myocardium of sham-operated rats or in the remote myocardium of rats with MI in PET/CT images 50-60 minutes post-injection (Figure 2A). However, there was higher ^68^Ga-NODAGA-exendin-4 uptake in the infarct region of the LV than remote myocardium at 3 days (SUV 0.67 ± 0.068 vs 0.42 ± 0.034, *P* = 0.026) and 1 week (SUV 0.59 ± 0.081 vs 0.43 ± 0.067, *P* < 0.001) after MI (Figure 2B). Pre-injection of unlabeled exendin-4 peptide 10 minutes before ^68^Ga-NODAGA-exendin-4 decreased the signal in the infarct region (SUV 0.39 ± 0.081, *P* = 0.0073 vs without pre-injection) to the same level as in the sham-operated rats at 1 week post-MI (Figure 2B). Maximum-intensity-projection PET/CT images of the whole thorax and SUV values in the organs adjacent to the heart are shown in Supplemental Figure 4 and Table 3. Tracer uptake in the chest wound caused by the operation was comparable to uptake in the infarcted region and partially reduced by pre-injection of unlabeled exendin-4 peptide.

**Figure 3:**
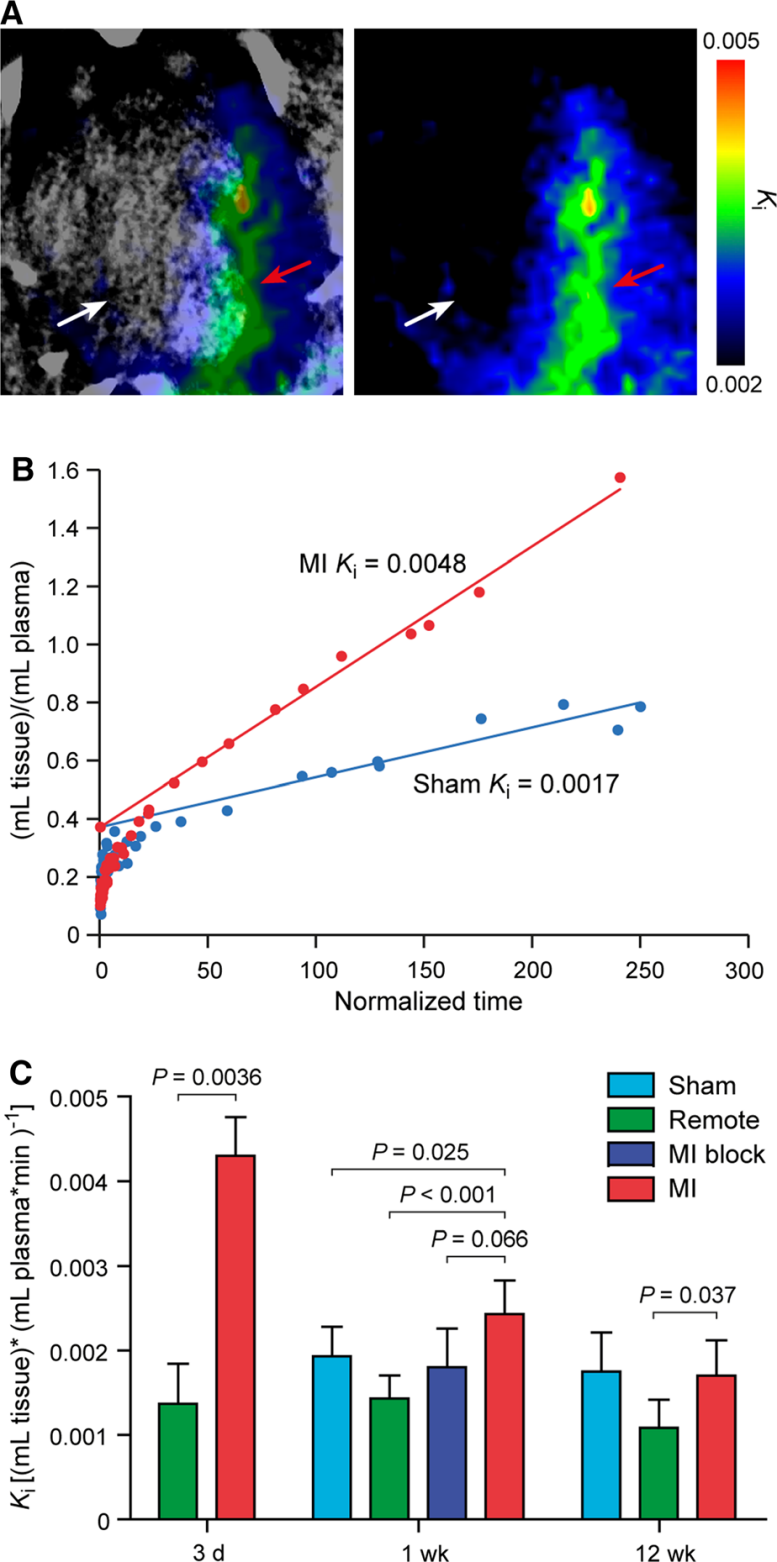
Kinetic analysis of ^68^Ga-NODAGA-exendin-4 uptake. **A** Horizontal long axis *K*_i_-generated positron emission tomography and corresponding contrast-enhanced computed tomography image showing clear and focal ^68^Ga-NODAGA-exendin-4 uptake in the anterior wall of the left ventricle 1 week after myocardial infarction (MI) (red arrows) compared with remote myocardium (white arrows). **B** Representative Patlak plots of the MI and sham-operated rat. The slope of the linear portion represents the tracer influx rate constant (*K*_i_), which refers to irreversible receptor binding and/or internalization of ^68^Ga-NODAGA-exendin-4. **C** Bars show the quantifications of *K*_i_ values. Values are mean ± SD; Student’s *t*-test for unpaired and paired (MI vs remote) measurements; 3 d n = 3, Sham 1 wk n = 7, MI 1 wk n = 7, MI block n = 3, Sham 12 wk n = 6, MI 12 wk n = 6. (*d*, day; *wk*, week)

Parametric *K*_i_-images and ROI-based Patlak plots confirmed ^68^Ga-NODAGA-exendin-4 uptake in the infarcted area, whereas the uptake was low in the remote myocardium or myocardium of sham-operated rats (Figure 3A, B). Net influx rate of ^68^Ga-NODAGA-exendin-4 was higher in the infarct region of the LV than remote myocardium at 3 days (*K*_i_ 0.0043 ± 0.00048 vs 0.0014 ± 0.00045, *P* = 0.0036), 1 week (*K*_i_ 0.0024 ± 0.00041 vs 0.0014 ± 0.0026, *P* < 0.001), and 12 weeks (*K*_i_ 0.0017 ± 0.00042 vs 0.0011 ± 0.00033, *P* = 0.037) after MI (Figure 3C). The net influx rate was decreased to the same level as in the sham-operated rats after pre-injection of unlabeled exendin-4 peptide (Figure 3C). *K*_i_ values correlated closely with SUV_mean_ (50-60 min) values (*r* = 0.53, *P* = 0.0034).

### ^68^Ga-NODAGA-exendin-4 Biodistribution and Autoradiography

Ex vivo biodistribution at 80 minutes post-injection showed markedly higher ^68^Ga-NODAGA-exendin-4 radioactivity in the infarct region of the LV than remote myocardium (SUV 0.43 ± 0.070 vs 0.065 ± 0.0078, *P* = 0.010) 1 week after MI. Furthermore, tracer uptake in the infarcted area was higher than the radioactivity in the blood (SUV 0.15 ± 0.033, *P* = 0.0089). Corresponding SUV values for pancreas and spleen were 0.096 ± 0.0088 and 0.15 ± 0.028, respectively.

**Figure 4:**
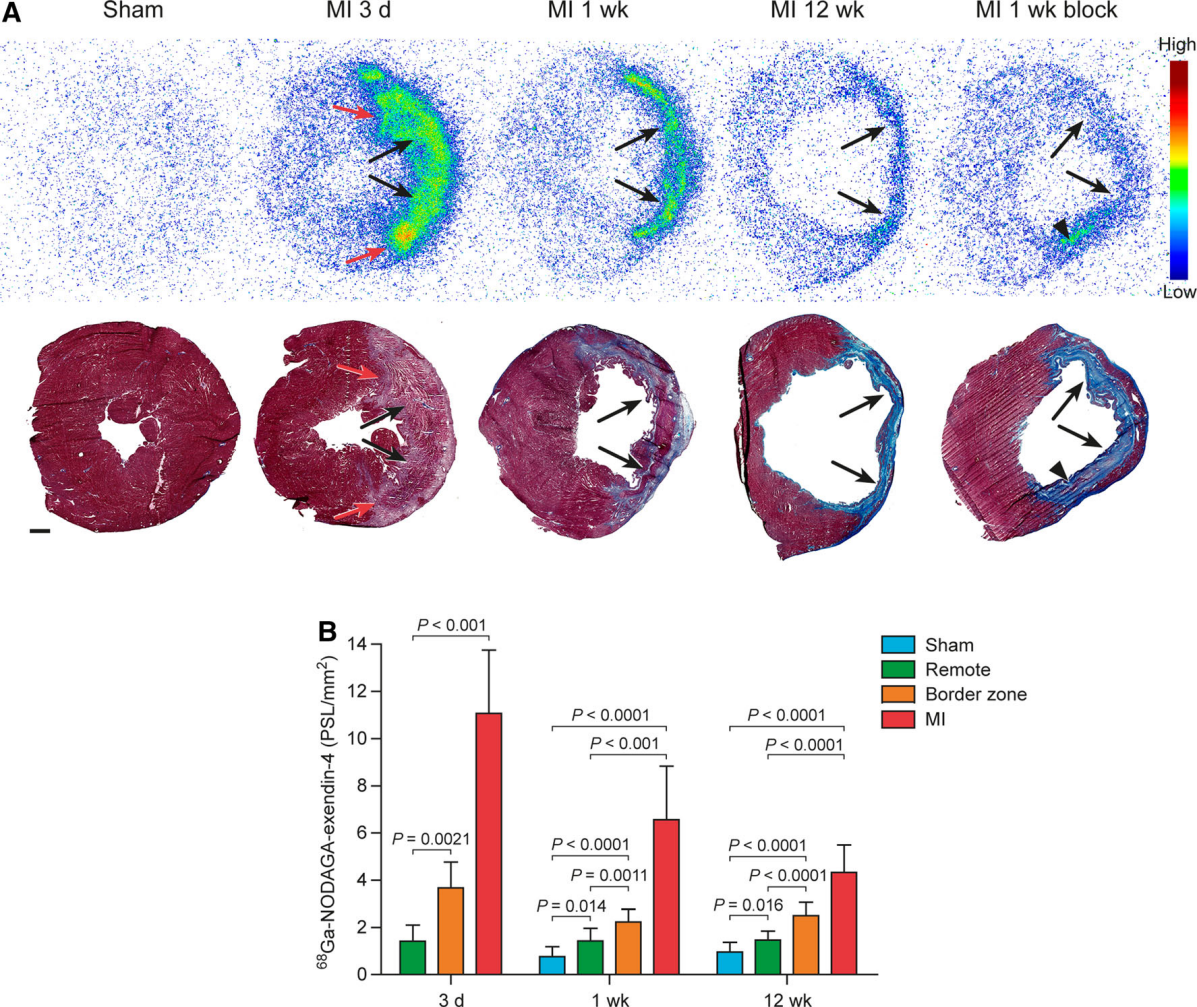
Ex vivo myocardial uptake of ^68^Ga-NODAGA-exendin-4. **A** Autoradiographs and corresponding Masson’s trichrome stainings of the left ventricle cross sections (scale bar 1 mm) showing increased ^68^Ga-NODAGA-exendin-4 uptake in the infarcted area (arrows) at 3 days, 1 week and 12 weeks after myocardial infarction (MI), whereas uptake is low after sham-operation or injection of unlabeled exendin-4 (MI 1 wk block). Red arrows indicate border zone and arrowhead non-specific residual activity in the area of necrotic myocyte debris. **B** Quantification of ^68^Ga-NODAGA-exendin-4 uptake as photo-stimulated luminescence per square millimeter (PSL/mm^2^, mean ± SD) in infarcted area, border zone, remote myocardium and myocardium of sham-operated rats. Student’s *t*-test for unpaired and paired (MI vs remote) measurements; 3 d n = 6, Sham 1 wk n = 9, MI 1 wk n = 7, Sham 12 wk n = 9, MI 12 wk n = 9. (*d*, day; *wk*, week)

Representative ^68^Ga-NODAGA-exendin-4 autoradiographs and quantitative results of ^68^Ga-NODAGA-exendin-4 uptake in different myocardial regions are presented in Figure 4 and Supplemental Table 4. Tracer uptake was low in the myocardium of sham-operated rats. There was focally increased ^68^Ga-NODAGA-exendin-4 uptake co-localizing with the MI scar in all rats after coronary ligation. Uptake of ^68^Ga-NODAGA-exendin-4 in the myocardium peaked 3 days after coronary ligation. Compared with the myocardium of sham-operated rats, ^68^Ga-NODAGA-exendin-4 uptake was 8.6-fold higher at 1 week and 4.5-fold higher at 12 weeks. In the MI border zone, ^68^Ga-NODAGA-exendin-4 uptake was 2.9- and 2.6-folds higher than in the myocardium of sham-operated rats at 1 and 12 weeks after MI. In the remote, non-infarcted myocardium, tracer uptake was also increased by 1.9- and 1.5-folds at 1 week and 12 weeks as compared with the myocardium of sham-operated rats.

After pre-injection of unlabeled exendin-4 peptide, there was marked reduction in tracer uptake in the infarcted area with some residual activity in the areas of necrotic myocyte debris. Quantitatively, pre-injection decreased the uptake by 33% (4.4 ± 1.4 PSL/mm^2^, *P* = 0.16 vs without pre-treatment) in the infarcted area, 51% (1.1 ± 0.050 PSL/mm^2^, *P* = 0.0078) in the border zone, and 39% (0.87 ± 0.15 PSL/mm^2^, *P* = 0.12) in the remote myocardium at 1 week post-MI.

### Localization of GLP-1R and Histological Correlations of ^68^Ga-NODAGA-exendin-4 Uptake

**Figure 5:**
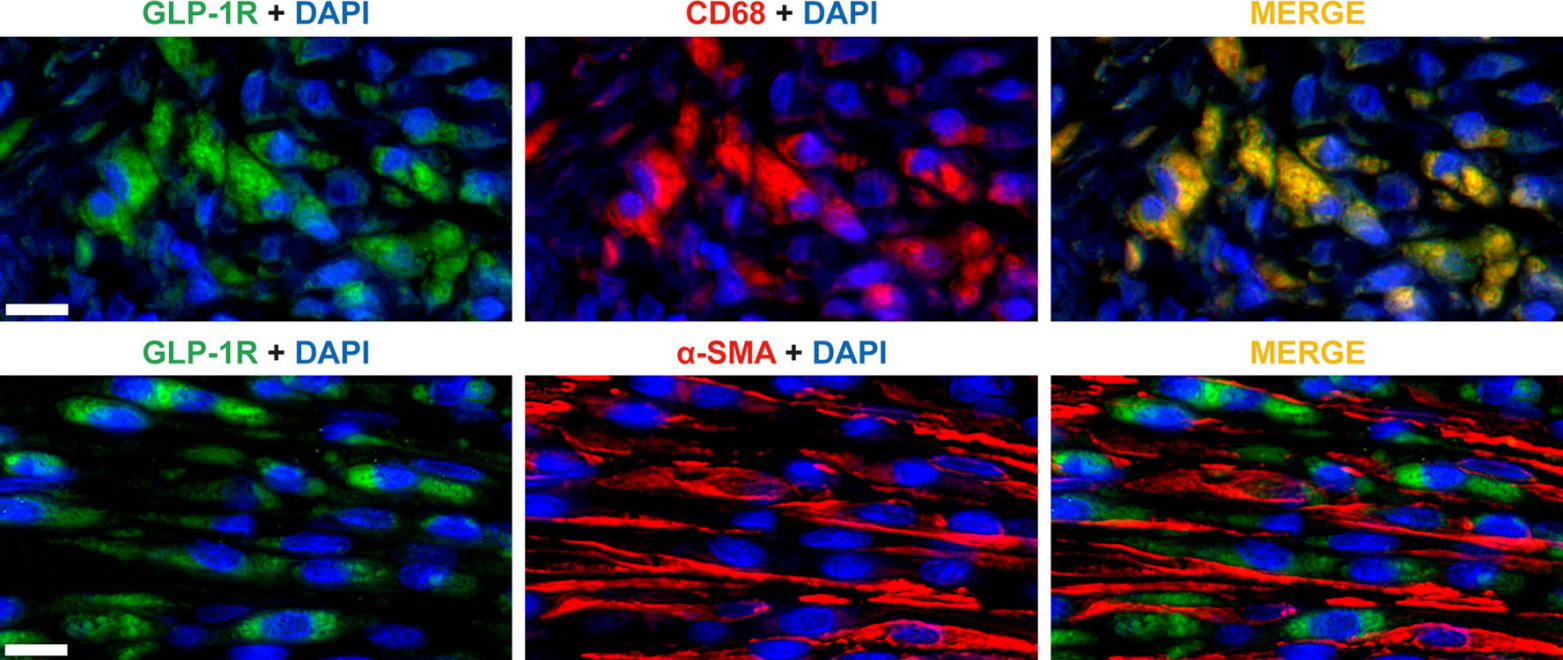
Localization of GLP-1R 1 week after coronary ligation. Double immunofluorescence staining for GLP-1R (green) and CD68-positive macrophages (red) showing complete co-localization (yellow, merge), whereas GLP-1R (green) do not co-localize (merge) with α-SMA-positive cells (red) in the infarcted area. Scale bar 10 μm. (*α-SMA*, alpha-smooth muscle actin; *GLP-1R*, glucagon-like peptide-1 receptor)

There was no staining with GLP-1R antibody in the myocardium of sham-operated rats. However, immunohistochemistry demonstrated GLP-1R-positive staining in the infarcted area and in the remote, non-infarcted myocardium 1 week after MI (Figures 1 and 5). In the infarcted area, GLP-1R-positive cells co-localized with the CD68-positive macrophages, but not with α-SMA staining (Figure 5).

**Figure 6:**
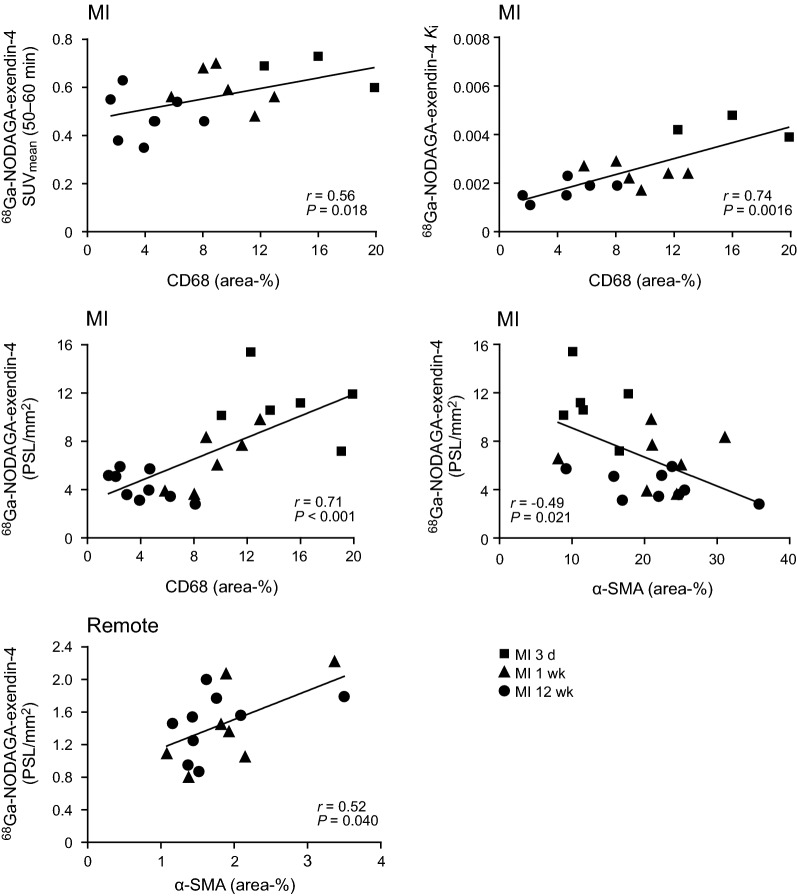
Histological correlations of ^68^Ga-NODAGA-exendin-4 uptake. Scatter plots showing positive correlation between CD68 staining and ^68^Ga-NODAGA-exendin-4 uptake in the infarcted area both by in vivo PET and autoradiography, whereas there is a negative correlation with α-SMA staining in the infarcted area. In the remote myocardium, ^68^Ga-NODAGA-exendin-4 uptake correlates positively with α-SMA staining. *r *= Spearman’s rank correlation coefficient. (*α-SMA*, alpha-smooth muscle actin; *d*, day; *MI*, myocardial infarction; *PSL/mm*^2^, photo-stimulated luminescence per square millimeter; *wk*, week)

The area of CD68 staining in the infarcted area correlated both with ^68^Ga-NODAGA-exendin-4 SUV_mean_ values (*r* = 0.56, *P* = 0.018) and *K*_i_ values in the infarcted area (*r* = 0.74, *P* = 0.0016) (Figure 6). In autoradiography, there was a good correlation between ^68^Ga-NODAGA-exendin-4 uptake and the area of CD68 staining in the infarcted area (*r* = 0.71, *P* < 0.001, pooled 3 days, 1 and 12 weeks). Although there was a negative correlation between ^68^Ga-NODAGA-exendin-4 uptake and area of α-SMA staining in the infarcted area (*r* = – 0.49, *P* = 0.021, pooled 3 days, 1 and 12 weeks), tracer uptake correlated with α-SMA-positivity in the remote myocardium from 1 to 12 weeks (*r* = 0.52, *P* = 0.040). (Figure 6) ^68^Ga-NODAGA-exendin-4 uptake did not correlate with the area of fibrosis in Masson’s trichrome staining in the infarcted area or in the remote myocardium.

## Discussion

We found that ^68^Ga-NODAGA-exendin-4 PET/CT detects an increase in myocardial GLP-1R expression after MI in rats. The level of ^68^Ga-NODAGA-exendin-4 uptake correlates with the amount of CD68-positive macrophages in the infarcted area and α-SMA-positive interstitial cells in the remote myocardium during the healing phase of MI.

Our findings with ^68^Ga-NODAGA-exendin-4 PET after permanent coronary occlusion are in line with a previous study showing uptake of ^18^F-FBEM-Cys^40^-exendin-4 during the first days (8 hours to 3 days) after myocardial ischemia-reperfusion injury.^16^ Our results extend the previous findings in that the ^68^Ga-NODAGA-exendin-4 uptake was detectable by PET/CT in the infarct region 1 week after MI when ex vivo analyses showed 9-fold higher uptake in the infarct region than in the myocardium of sham-operated rats and 3-fold higher uptake than in the blood. Previous in vitro studies with related NODAGA-exendin-4 radiotracers have suggested 20-35% internalization.^19^ In line with that, kinetic modeling by graphical Patlak analysis supported irreversible ^68^Ga-NODAGA-exendin-4 uptake in the infarcted area. The observed irreversible compartment can be due to irreversible receptor binding and/or internalization, since Patlak analysis calculates the net influx rate of irreversible uptake.^20^ Furthermore, unlabeled exendin-4 peptide significantly reduced uptake in the infarcted area indicating specific receptor-mediated uptake. Tracer uptake in the chest wound was also partially reduced by pre-injection of unlabeled exendin-4 peptide at this time, which is consistent with the role of exendin-4 in wound healing.^21^ Compared with ^18^F-FBEM-Cys^40^-exendin-4, the lung uptake of ^68^Ga-NODAGA-exendin-4 was lower. This may be due to differences in tracer structure.^19^ Furthermore, ex vivo analyses showed that up-regulation of GLP-1R continued in the infarct scar as long as 12 weeks after MI and also in the remote, non-infarcted myocardium from 1 to 12 weeks after MI. We also demonstrate that the uptake of ^68^Ga-NODAGA-exendin-4 correlated with the area of CD68-positive macrophages in the infarcted area, and with the area of α-SMA-positive interstitial cells in the remote myocardium after MI.

The GLP-1R may be an interesting target for cardiac imaging, because it has been proposed to play a role in repair of an ischemic myocardial injury and post-MI remodeling.^2^,^8^,^12^ Consistent with the role of GLP-1R signaling in wound healing,^21^ GLP-1R activation induced differentiation of human macrophages into anti-inflammatory or reparative M2 phenotype via STAT3 activation,^10^ increased the amount of M2 macrophages after MI^11^ and attenuated myocardial inflammation and interstitial fibrosis after MI by modulation of Akt/GSK-3β and Smad2/3 signaling in macrophages.^8^ However, the relationship between GLP-1R expression and mechanisms associated with healing of MI remains unclear. In a previous study, no co-localization of GLP-1R with CD11b neutrophils was observed after MI,^16^ but GLP-1R expression has been found in macrophages in atherosclerotic lesions^22^ and in murine bone marrow derived macrophages.^8^ We found that macrophages expressed GLP-1R and the amount of macrophages correlated with ^68^Ga-NODAGA-exendin-4 uptake in the infarct region both by in vivo PET and autoradiography analyses. These results indicate up-regulation of GLP-1R being potentially associated with the activation of repair mechanisms after ischemic injury that may be evaluated using ^68^Ga-NODAGA-exendin-4 PET.

There was a moderate increase of ^68^Ga-NODAGA-exendin-4 uptake in the remote myocardium 1 and 12 weeks after MI that correlated with α-SMA-positive staining. In recent studies, GLP-1R expression was not detected in ventricular myocytes,^23^ cardiac fibroblasts^3^,^8^,^23^ and coronary artery smooth muscle cells.^23^ In line with that, we did not find co-localization of GLP-1R and α-SMA-positive staining in the myocardium after MI and there was a negative correlation between ^68^Ga-NODAGA-exendin-4 uptake and α-SMA-positive staining in the infarcted area. The low content of macrophages in the remote myocardium did not allow analysis of correlation to ^68^Ga-NODAGA-exendin-4 uptake. However, this does not mean that the number of macrophages is not increased in the remote region after MI, since conventional histology is less sensitive to small changes. Thus, the substrate of ^68^Ga-NODAGA-exendin-4 uptake in the remote myocardium remained unexplained. Previous studies indicate that GLP-1 may attenuate myocardial extracellular matrix remodeling in the absence of direct actions on cardiac fibroblast differentiation via angiotensin production or macrophage-dependent mechanisms.^8^,^9^,^24^

## Limitations

The determinants of compensatory increase in cardiac GLP-1R expression are incompletely understood^24^ and thus, the possible effects of type 2 diabetes, anti-inflammatory therapy, or therapies activating GLP-1R signaling on cardiac GLP-1R expression and ^68^Ga-NODAGA-exendin-4 uptake should be studied. The incremental value of ^68^Ga-NODAGA-exendin-4 PET in predicting subsequent cardiac function and remodeling after MI remain to be tested prospectively. Only male rats were used in the present study and it has to be studied whether ^68^Ga-NODAGA-exendin-4 uptake is different in females after MI. The identity of observed radio-metabolites of ^68^Ga-NODAGA-exendin-4 or whether they cross into the tissues remains to be studied. Limited spatial resolution of the small-animal ^68^Ga PET imaging, partial volume effects, and spill over from blood or adjacent tissues may explain lower infarct-to-remote myocardium ratios by in vivo ^68^Ga-NODAGA-exendin-4 PET/CT than observed in ex vivo analyses and cause inaccuracy in quantification of cardiac tracer uptake in vivo. Thus, a large animal model or a study in patients with MI with a tracer suitable for a clinical study^14^,^15^ would be needed to address the above questions.

## New Knowledge Gained

PET imaging with a specific GLP-1R-targeted radioligand, ^68^Ga-NODAGA-exendin-4, shows an increase in myocardial GLP-1R expression correlating with the presence of macrophages during healing phase of MI in rats.

## Conclusions

Our results show that ^68^Ga-NODAGA-exendin-4 PET detects up-regulated cardiac GLP-1R expression after MI in rats. Furthermore, ^68^Ga-NODAGA-exendin-4 PET may be useful to elucidate the role of GLP-1R expression in macrophages involved in myocardial repair after an ischemic injury.

## Electronic supplementary material

Below is the link to the electronic supplementary material.

Supplementary material 1 (DOCX 1583 kb)

Supplementary material 2 (PPTX 1731 kb)

## Abbreviations

α-SMA: Alpha-smooth muscle actin
GLP-1R: Glucagon-like peptide-1 receptor
LCA: Left coronary artery
LV: Left ventricle
MI: Myocardial infarction
PET/CT: Positron emission tomography/computed tomography
ROI: Region of interest
SUV: Standardized uptake value
