# Supplementary material for: Glucagon-like peptide-1 receptor expression after myocardial infarction: Imaging study using 68Ga-NODAGA-exendin-4 positron emission tomography
Source: J Nucl Cardiol. 2018 Dec 13;27(6):2386–97. doi: 10.1007/s12350-018-01547-1 (PMC7749060; doi:10.1007/s12350-018-01547-1)
Supplement: Supplementary file 1 — Supplementary material 1 (DOCX 1583 kb) [file 12350_2018_1547_MOESM1_ESM.docx]

**Journal of Nuclear Cardiology**

# **SUPPLEMENTARY DATA**

**Glucagon-like peptide-1 receptor expression after myocardial infarction:**

**Imaging study using ^68^Ga-NODAGA-exendin-4 positron emission tomography**

Mia Ståhle, MSc,^a^ Ville Kytö, MD, PhD,^b,c^ Max Kiugel, MSc,^a^ Heidi Liljenbäck, MSc,^a,d^ Olli Metsälä, MSc,^a^ Meeri Käkelä, MSc,^a^ Xiang-Guo Li, PhD,^a,e^ Vesa Oikonen, MSc,^a^ Pekka Saukko, MD, PhD,^f^ Pirjo Nuutila, MD, PhD,^a,g,h^ Juhani Knuuti, MD, PhD,^a,h^ Anne Roivainen, PhD,^a,d^ and Antti Saraste, MD, PhD^a,b,h^

^a^ Turku PET Centre, University of Turku, Turku, Finland

^b^ Heart Center, Turku University Hospital, Turku, Finland

^c^ Research Centre of Applied and Preventive Cardiovascular Medicine, University of Turku, Turku, Finland

^d^ Turku Center for Disease Modeling, University of Turku, Turku, Finland

^e^ Turku PET Centre, Åbo Akademi University, Turku, Finland

^f^ Department of Pathology and Forensic Medicine, University of Turku, Turku, Finland

^g^ Department of Endocrinology, Turku University Hospital, Turku, Finland

^h^ Turku PET Centre, Turku University Hospital, Turku, Finland

**Address for correspondence** Antti Saraste**,** Turku PET Centre, Kiinamyllynkatu 4-8, FI-20520, Turku, Finland. Tel: +35823130083, Fax: +35822318191, E-mail: antti.saraste@utu.fi.

# **SUPPLEMENTAL METHODS**


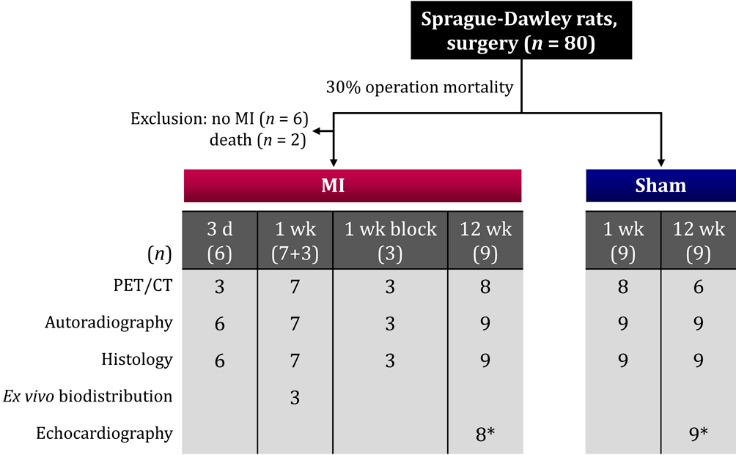


**Supplemental Figure 1.** A flow diagram of studies. Numbers and subgroups of animals used in the study and analyses. After coronary ligation, mortality was approximately 30% during the first two days. Thereafter, only two rats died spontaneously during the follow-up. Six rats were excluded because there was no MI (<4% of the endocardial circumference) despite ligation. For technical reasons, PET/CT was not performed in all rats. Three rats with MI were used only for competition experiments at 1 week (1 wk block). *repeated measurements at 1 and 12 weeks. (*d*, day; *MI*, myocardial infarction; *n*, number; *PET/CT*, positron emission tomography/computed tomography; *wk*, week).

## **Echocardiography**

In a subset of rats (Supplemental Figure 1), the function and size of the LV were repeatedly assessed by echocardiography at 1 and 12 weeks after coronary ligation or the sham-operation. Echocardiography was performed using a dedicated small animal Doppler ultrasound device (Vevo 2100, VisualSonics, Inc., Toronto, ON, Canada) and a linear 13-24 MHz (MS250) transducer. The LV end-diastolic diameter, end-systolic diameter and thickness of the interventricular septum and posterior wall were measured in M-mode parasternal long-axis views. Fractional shortening and LV mass were calculated. Co-variance analysis for repeated measurements (ANCOVA), adjusted by the baseline value, was used to compare echocardiographic data.

## **Analysis of Autoradiographs and Histological Sections**

The average ^68^Ga-NODAGA-exendin-4 accumulation was measured as photo-stimulated luminescence per square millimeter (PSL/mm^2^) in 4 to 5 sections per heart in the infarcted area, the border zone myocardium consisting of 3 to 5 myocyte layers adjacent to the infarct, and the remote myocardium in the septum using Tina 2.1 software (Raytest Isotopenmessgeräte GmbH, Straubenhardt, Germany). Background radiation was subtracted from the image data and the results for each rat were decay corrected to injection time and exposure time, and normalized for injected radioactivity dose per animal weight in order to compare absolute PSL/mm^2^ values between animals.

Slides were scanned with a digital slide scanner (Pannoramic MIDI for immunofluorescence; 3DHistech Ltd., Budapest, Hungary) and analyzed by Image-J v.1.46 software (National Institutes of Health, Bethesda, MD, USA). The MI size was measured as the average percentage of LV circumference as described previously^1^. Percentages of myocardium positive for CD68, α-SMA or collagen within MI region or remote myocardium were measured in four sections per heart using specific color threshold values for each staining. These percentages were correlated with the ^68^Ga-NODAGA-exendin-4 accumulation in autoradiography images of the same areas in parallel sections.

**Supplemental Table 1.** Primary antibodies and detection methods used for histology.

|  |  |  |  |  |  |
| --- | --- | --- | --- | --- | --- |
| **Antibody** | **Clone** | **Dilution** | | **Manufacturer** | **Detection** |
|  |  | **Cryo** | **Paraffin** |  |  |
| **Immunohistochemistry** | |  |  |  |  |
| GLP-1R | Polyclonal rabbit anti-rat GLP-1R, |  | 1:500 | Abcam | Vectastain ABC-HRP kit (Vector Laboratories, |
|  | ab39072 |  |  | Cambridge, UK | Burlingame, CA, USA) + chromogen (DAB, Dako K3468) |
| CD68 | Monoclonal mouse anti-rat CD68, | 1:10 000 |  | AbD Serotec | Vectastain ABC-HRP kit (Vector Laboratories, |
|  | MCA341R |  |  | Munich, Germany | Burlingame, CA, USA) + chromogen (DAB, Dako K3468) |
| α-SMA | Monoclonal mouse anti-rat α-SMA, | 1:20 000 |  | Sigma-Aldrich | Vectastain ABC-HRP kit (Vector Laboratories, |
|  | A5228 |  |  | St. Louis, MO, USA | Burlingame, CA, USA) + chromogen (DAB, Dako K3468) |
| **Immunofluorescence** | |  |  |  |  |
| GLP-1R* | Polyclonal rabbit anti-rat GLP-1R, |  | 1:500 | Abcam | Donkey anti-rabbit Alexa 488, A21206, 1:200 |
|  | ab39072 |  |  | Cambridge, UK | (Invitrogen, Carlsbad, CA, USA) |
| CD68 | Monoclonal mouse anti-rat CD68, |  | 1:500 | AbD Serotec | Goat anti-mouse Alexa 594, 982335, 1:200 |
|  | MCA341R |  |  | Munich, Germany | (Invitrogen, Carlsbad, CA, USA) |
| α-SMA | Monoclonal mouse anti-rat α-SMA, |  | 1:2000 | Sigma-Aldrich | Goat anti-mouse Alexa 594, 982335, 1:200 |
|  | A5228 |  |  | St. Louis, MO, USA | (Invitrogen, Carlsbad, CA, USA) |
|  |  |  |  |  | Mounting medium: ProLong TM Gold antifade reagent |
|  |  |  |  |  | with DAPI, P36935 (Invitrogen, Carlsbad, CA, USA) |
| *Double immunofluorescence with CD68 or α-SMA. | | |  |  |  |
| *GLP-1R*, glucagon-like peptide-1 receptor; *α-SMA*, alpha-smooth muscle actin. | | | | |  |
|  |  |  |  |  |  |

# **SUPPLEMENTAL RESULTS**

## **Echocardiography**

The echocardiographic data in Supplemental Table 2 show that fractional shortening was lower after MI than the sham-operation both at week 1 and week 12 (*P* < 0.0001). Compared with week 1, the LV diastolic and systolic diameters showed a greater increase in rats with MI than sham-operation after 12 weeks (26 ± 16% vs 6.8 ± 8.5%, *P* < 0.0001 and 32 ± 17% vs 19 ± 16%, *P* = 0.046, respectively) indicating significant post-MI LV remodeling.

**Supplemental Table 2.** Echocardiographic data at 1 and 12 weeks.

|  |  |  |  |  |  |  |  |  |  |  |  |  |  |
| --- | --- | --- | --- | --- | --- | --- | --- | --- | --- | --- | --- | --- | --- |
|  |  | **Week 1** | | |  | **Week 12** | | |  | **Absolute change** | | | *P*-value† |
| **LVDD (mm)** | Sham | 8.7 | ± | 0.40 |  | 9.2 | ± | 0.43 |  | 0.57 | ± | 0.70 | <0.0001 |
|  | MI | 11 | ± | 1.1 |  | 13 | ± | 1.1 |  | 2.7 | ± | 1.4 |  |
|  | *P*-value* | <0.001 | | |  | <0.0001 | | |  |  |  |  |  |
|  |  |  |  |  |  |  |  |  |  |  |  |  |  |
| **LVSD (mm)** | Sham | 4.5 | ± | 0.40 |  | 5.3 | ± | 0.55 |  | 0.83 | ± | 0.61 | 0.046 |
|  | MI | 8.7 | ± | 1.2 |  | 11 | ± | 1.5 |  | 2.7 | ± | 1.2 |  |
|  | *P*-value* | <0.0001 | | |  | <0.0001 | | |  |  |  |  |  |
|  |  |  |  |  |  |  |  |  |  |  |  |  |  |
| **IVST (mm)** | Sham | 0.86 | ± | 0.13 |  | 0.81 | ± | 0.10 |  | -0.044 | ± | 0.12 | 0.63 |
|  | MI | 0.63 | ± | 0.16 |  | 0.63 | ± | 0.21 |  | 0 | ± | 0.15 |  |
|  | *P*-value* | 0.0052 | | |  | 0.027 | | |  |  |  |  |  |
|  |  |  |  |  |  |  |  |  |  |  |  |  |  |
| **PWT (mm)** | Sham | 0.90 | ± | 0.12 |  | 0.93 | ± | 0.13 |  | 0.033 | ± | 0.17 | 0.18 |
|  | MI | 0.83 | ± | 0.070 |  | 0.81 | ± | 0.17 |  | -0.013 | ± | 0.17 |  |
|  | *P*-value* | 0.15 | | |  | 0.11 | | |  |  |  |  |  |
|  |  |  |  |  |  |  |  |  |  |  |  |  |  |
| **FS (%)** | Sham | 48 | ± | 5.1 |  | 42 | ± | 5.6 |  | -5.8 | ± | 0.055 | 0.29 |
|  | MI | 17 | ± | 5.0 |  | 13 | ± | 5.1 |  | -3.7 | ± | 0.027 |  |
|  | *P*-value* | <0.0001 | | |  | <0.0001 | | |  |  |  |  |  |
|  |  |  |  |  |  |  |  |  |  |  |  |  |  |
| **LV mass (g)** | Sham | 0.48 | ± | 0.10 |  | 0.54 | ± | 0.086 |  | 0.052 | ± | 0.15 | 0.0033 |
|  | MI | 0.56 | ± | 0.14 |  | 0.83 | ± | 0.20 |  | 0.27 | ± | 0.20 |  |
|  | *P*-value* | 0.23 | | |  | 0.0011 | | |  |  |  |  |  |
|  |  |  |  |  |  |  |  |  |  |  |  |  |  |
| **LV mass (g)/** | Sham | 0.0017 | ± | 0.00034 |  | 0.0012 | ± | 0.00018 |  | -0.00054 | ± | 0.00041 | 0.0059 |
| **Body weight (g)** | MI | 0.0020 | ± | 0.00049 |  | 0.0017 | ± | 0.00039 |  | -0.00025 | ± | 0.00050 |  |
|  | *P*-value* | 0.17 | | |  | 0.0018 | | |  |  |  |  |  |
|  |  |  |  |  |  |  |  |  |  |  |  |  |  |
| **HR (beats/min)** | Sham | 340 | ± | 31 |  | 340 | ± | 17 |  | 1.0 | ± | 31 | 0.0025 |
|  | MI | 330 | ± | 31 |  | 310 | ± | 14 |  | -18 | ± | 32 |  |
|  | *P*-value* | 0.50 | | |  | 0.0014 | | |  |  |  |  |  |
| Values are mean ± SD. *Student's *t*-test for unpaired measurements. †Co-variance analysis adjusted by | | | | | | | | | | | | | |
| week 1 measurement. *FS*, fractional shortening; *HR*, heart rate; *LV*, left ventricle; | | | | | | | | | | |  |  |  |
| *IVST*, interventricular septal thickness; *LVDD* and *LVSD*, left ventricular diastolic and systolic diameter, | | | | | | | | | | | | | |
| respectively; *MI*, myocardial infarction; *PWT*, posterior wall thickness. | | | | | | | | | |  |  |  |  |
|  |  |  |  |  |  |  |  |  |  |  |  |  |  |

**

**

**Supplemental Figure 2.** Structure of [Nle^14^,Lys^40^(Ahx-NODAGA)NH_2_]-exendin-4 peptide. The peptide sequence is HGEGTFTSDLSKQ**B**EEEAVRLFIEWLKNGGPSSGAPPPSK(**X**-NODAGA)NH_2_, where **B** = methionine is replaced with norleucine, **X** = aminohexanoic acid, and NODAGA = 1,4,7-triazacyclononane-1-glutamic acid-4,7-diacetic acid. Chemical formula is C_212_H_331_N_56_O_69_ and molecular weight 4765.4 g/mol.

**
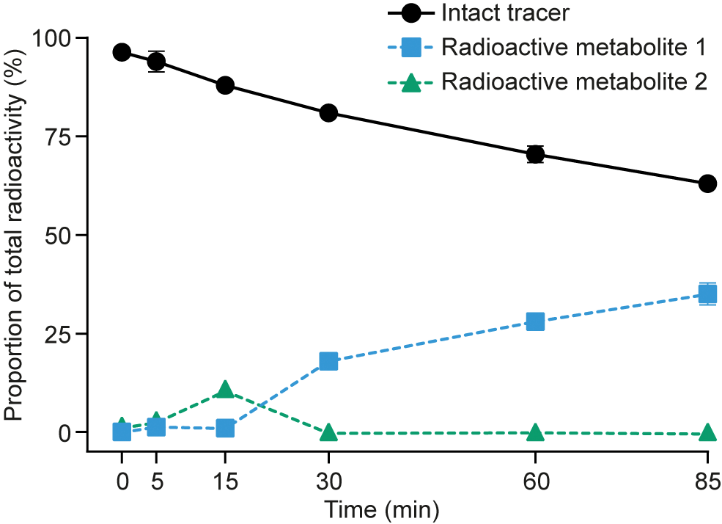
**

**Supplemental Figure 3.** In vivo stability of ^68^Ga-NODAGA-exendin-4 tracer. Curves represent the proportion (%) of unchanged ^68^Ga-NODAGA-exendin-4 and radioactive metabolites in rat plasma 12 weeks after the sham-operation (n= 3, mean ± SD) as a function of time after intravenous injection.


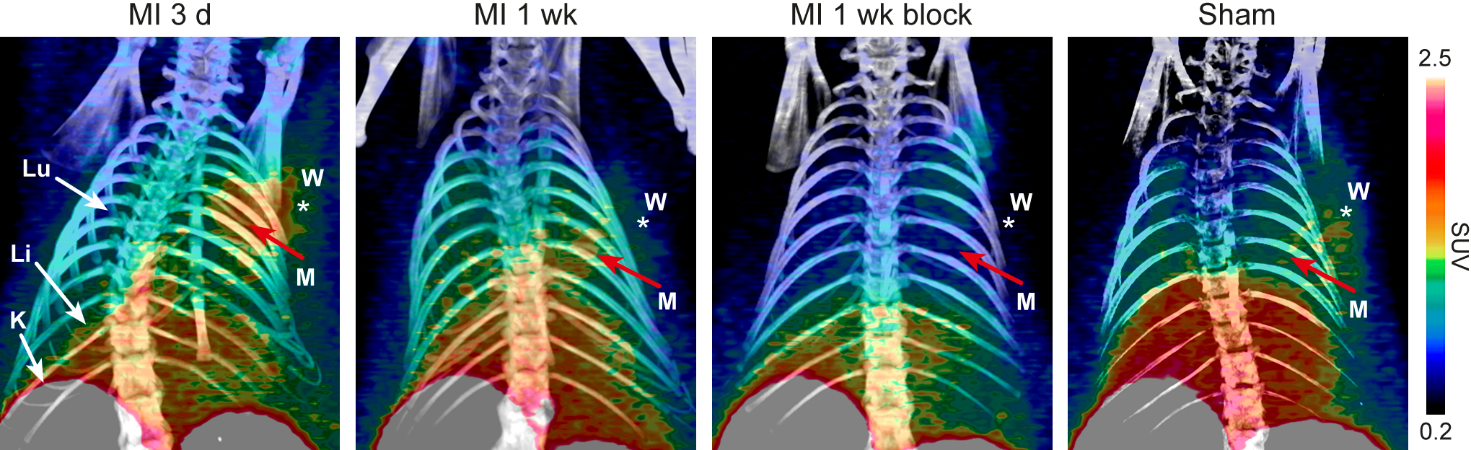


**Supplemental Figure 4.** ^68^Ga-NODAGA-exendin-4 maximum-intensity-projection positron emission tomography/computed tomography images of the whole thorax 50 to 60 minutes after injection. Red arrows indicate infarcted region or corresponding anterior wall in sham-operated rats. (*d*, day; *Li*, liver; *Lu*, lung; *M*, myocardium; *K*, kidney; *SUV*, standardized uptake value; *W*, chest wound; *wk*, week).

**Supplemental Table 3.** In vivo quantification of ^68^Ga-NODAGA-exendin-4 uptake.

|  |  |  |  |  |  |  |  |  |  |  |  |  |  |  |  |  |  |  |  |  |
| --- | --- | --- | --- | --- | --- | --- | --- | --- | --- | --- | --- | --- | --- | --- | --- | --- | --- | --- | --- | --- |
|  | **3 days** | | |  | **1 week** | | | | | | | | |  | **12 weeks** | | | | | |
|  | **MI** | | |  | **Sham** | | | **MI** | | | **MI block** | | |  | **Sham** | | | **MI** | | |
| **Myocardium** | 0.67 | ± | 0.068 |  | 0.42 | ± | 0.12 | 0.59 | ± | 0.081* | 0.39 | ± | 0.081^†^ |  | 0.41 | ± | 0.049 | 0.48 | ± | 0.092 |
| **Liver** | 0.60 | ± | 0.074 |  | 1.5 | ± | 0.42 | 1.8 | ± | 0.74 | 0.66 | ± | 0.10^†^ |  | 2.3 | ± | 0.50^‡‡^ | 1.7 | ± | 1.1 |
| **Lung** | 0.39 | ± | 0.10 |  | 0.57 | ± | 0.14 | 0.56 | ± | 0.24 | 0.25 | ± | 0.031 |  | 0.55 | ± | 0.14 | 0.59 | ± | 0.14 |
| **Kidney** | 38 | ± | 7.6 |  | 32 | ± | 6.2 | 31 | ± | 5.1 | 38 | ± | 0.40 |  | 40 | ± | 3.6^‡‡^ | 38 | ± | 5.3 |
| **Muscle** | 0.16 | ± | 0.025 |  | 0.12 | ± | 0.017 | 0.13 | ± | 0.026 | 0.089 | ± | 0.021 |  | 0.11 | ± | 0.028 | 0.12 | ± | 0.040 |
| **Wound** | 0.69 | ± | 0.070 |  | 0.56 | ± | 0.10 | 0.59 | ± | 0.064 | 0.50 | ± | 0.034^†^ |  | 0.37 | ± | 0.12^‡‡^ | 0.40 | ± | 0.14^$^ |
| Results are expressed as standardized uptake values (SUV_mean_ 50-60 min ± SD); Student’s unpaired *t*-test and | | | | | | | | | | | | | | | | | | | | |
| ANOVA for comparisons of MI 3d, 1wk and 12 wk; *P<0.05 MI vs Sham,^†^P<0.05 MI block vs MI, | | | | | | | | | | | | | | | | | | | | |
| ^‡‡^P<0.05 Sham 1wk vs Sham 12wk, ^$^P<0.01 MI 12 wk vs 3 d and 1 wk. *MI*, myocardial infarction; | | | | | | | | | | | | | | | | | | | | |
| *Myocardium*, infarcted region or corresponding anterior wall in sham-operated rats. | | | | | | | | | | | | | | | | | | | | |
|  |  |  |  |  |  |  |  |  |  |  |  |  |  |  |  |  |  |  |  |  |

**Supplemental Table 4.** Autoradiography of ^68^Ga-NODAGA-exendin-4 uptake in myocardial tissue sections after coronary ligation or sham-operation.

|  |  |  |  |  |  |  |  |  |  |  |  |
| --- | --- | --- | --- | --- | --- | --- | --- | --- | --- | --- | --- |
|  | **3 days** | | |  | **1 week** | | |  | **12 weeks** | | |
| **Infarct area** | 11±2.7 | | |  | 6.6±2.3 | | |  | 4.3±1.2 | | |
| *P* vs Remote | <0.001 | | |  | <0.001 | | |  | <0.0001 | | |
| *P* vs Sham |  |  |  |  | <0.0001 | | |  | <0.0001 | | |
|  |  |  |  |  |  |  |  |  |  |  |  |
| **Border zone** | 3.7±1.1 | | |  | 2.2±0.54 | | |  | 2.5±0.56 | | |
| *P* vs Remote | 0.0021 | | |  | 0.0011 | | |  | <0.0001 | | |
| *P* vs Sham |  |  |  |  | <0.0001 | | |  | <0.0001 | | |
|  |  |  |  |  |  |  |  |  |  |  |  |
| **Remote** | 1.4±0.68 | | |  | 1.4±0.53 | | |  | 1.5±0.38 | | |
| *P* vs Sham |  |  |  |  | 0.014 | | |  | 0.016 | | |
|  |  |  |  |  |  |  |  |  |  |  |  |
| **Sham** |  |  |  |  | 0.76±0.42 | | |  | 0.96±0.41 | | |
| Results are expressed as photo-stimulated luminescence per | | | | | | | | | | | |
| square millimeter (PSL/mm^2^), mean ± SD; Student’s *t-*test for paired (*P* vs Remote) and unpaired (*P* vs Sham) data. | | | | | | | | | | | |
|  |  |  |  |  |  |  |  |  |  |  |  |

**

**

**Supplemental Figure 5.** Correlations between echocardiographic parameters and ^68^Ga-NODAGA-exendin-4 uptake. At 12 weeks, ^68^Ga-NODAGA-exendin-4 uptake in the remote myocardium (autoradiography) correlates with myocardial infarction (MI) size, whereas there is an inverse correlation with fractional shortening (FS). There is also a tendency towards positive correlation between tracer uptake and left ventricle diastolic and systolic diameters (LVDD and LVSD, respectively). *r* = Spearman's rank correlation coefficient. (*PSL/mm^2^*, photo-stimulated luminescence per square millimeter).

# **REFERENCES**

1. Kiugel M, Dijkgraaf I, Kytö V, Helin S, Liljenbäck H, Saanijoki T, et al. Dimeric [^68^Ga]DOTA-RGD peptide targeting α_v_β_3_ integrin reveals extracellular matrix alterations after myocardial infarction. Mol Imaging Biol. 2014;16:793–801.
