# Supplementary material for: Glucagon-like peptide-1 receptor expression after myocardial infarction: Imaging study using 68Ga-NODAGA-exendin-4 positron emission tomography
Source: J Nucl Cardiol. 2018 Dec 13;27(6):2386–97. doi: 10.1007/s12350-018-01547-1 (PMC7749060; doi:10.1007/s12350-018-01547-1)
Supplement: Supplementary file 2 — Supplementary material 2 (PPTX 1731 kb) [file 12350_2018_1547_MOESM2_ESM.pptx]

## Slide 1
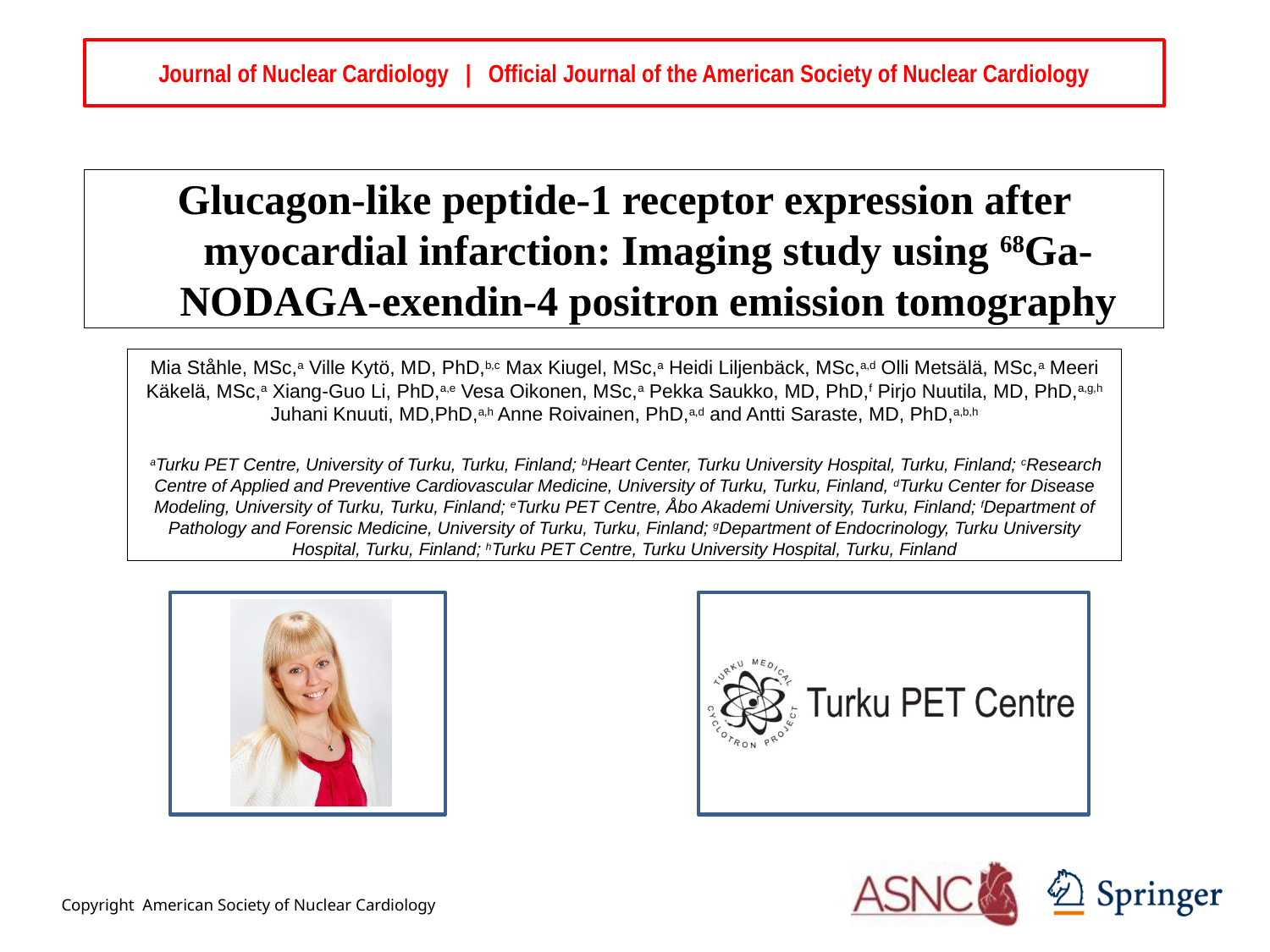

Journal of Nuclear Cardiology | Official Journal of the American Society of Nuclear Cardiology
# Glucagon-like peptide-1 receptor expression after myocardial infarction: Imaging study using 68Ga-NODAGA-exendin-4 positron emission tomography
Mia Ståhle, MSc,a Ville Kytö, MD, PhD,b,c Max Kiugel, MSc,a Heidi Liljenbäck, MSc,a,d Olli Metsälä, MSc,a Meeri Käkelä, MSc,a Xiang-Guo Li, PhD,a,e Vesa Oikonen, MSc,a Pekka Saukko, MD, PhD,f Pirjo Nuutila, MD, PhD,a,g,h Juhani Knuuti, MD,PhD,a,h Anne Roivainen, PhD,a,d and Antti Saraste, MD, PhD,a,b,h
 aTurku PET Centre, University of Turku, Turku, Finland; bHeart Center, Turku University Hospital, Turku, Finland; cResearch Centre of Applied and Preventive Cardiovascular Medicine, University of Turku, Turku, Finland, dTurku Center for Disease Modeling, University of Turku, Turku, Finland; eTurku PET Centre, Åbo Akademi University, Turku, Finland; fDepartment of Pathology and Forensic Medicine, University of Turku, Turku, Finland; gDepartment of Endocrinology, Turku University Hospital, Turku, Finland; hTurku PET Centre, Turku University Hospital, Turku, Finland
Copyright American Society of Nuclear Cardiology

## Slide 2
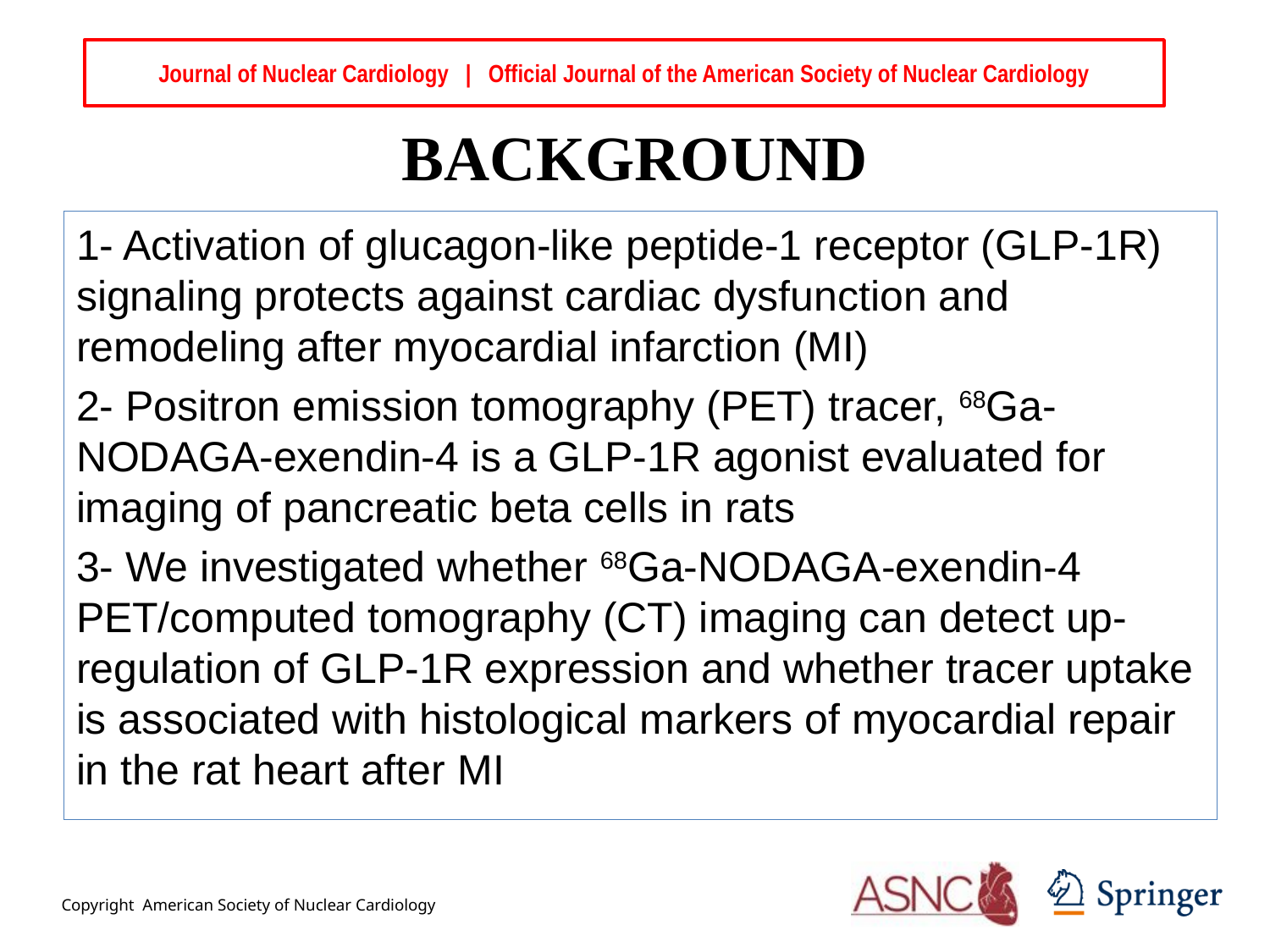

Journal of Nuclear Cardiology | Official Journal of the American Society of Nuclear Cardiology
# BACKGROUND
1- Activation of glucagon-like peptide-1 receptor (GLP-1R) signaling protects against cardiac dysfunction and remodeling after myocardial infarction (MI)
2- Positron emission tomography (PET) tracer, 68Ga-NODAGA-exendin-4 is a GLP-1R agonist evaluated for imaging of pancreatic beta cells in rats
3- We investigated whether 68Ga-NODAGA-exendin-4 PET/computed tomography (CT) imaging can detect up-regulation of GLP-1R expression and whether tracer uptake is associated with histological markers of myocardial repair in the rat heart after MI
Copyright American Society of Nuclear Cardiology

## Slide 3
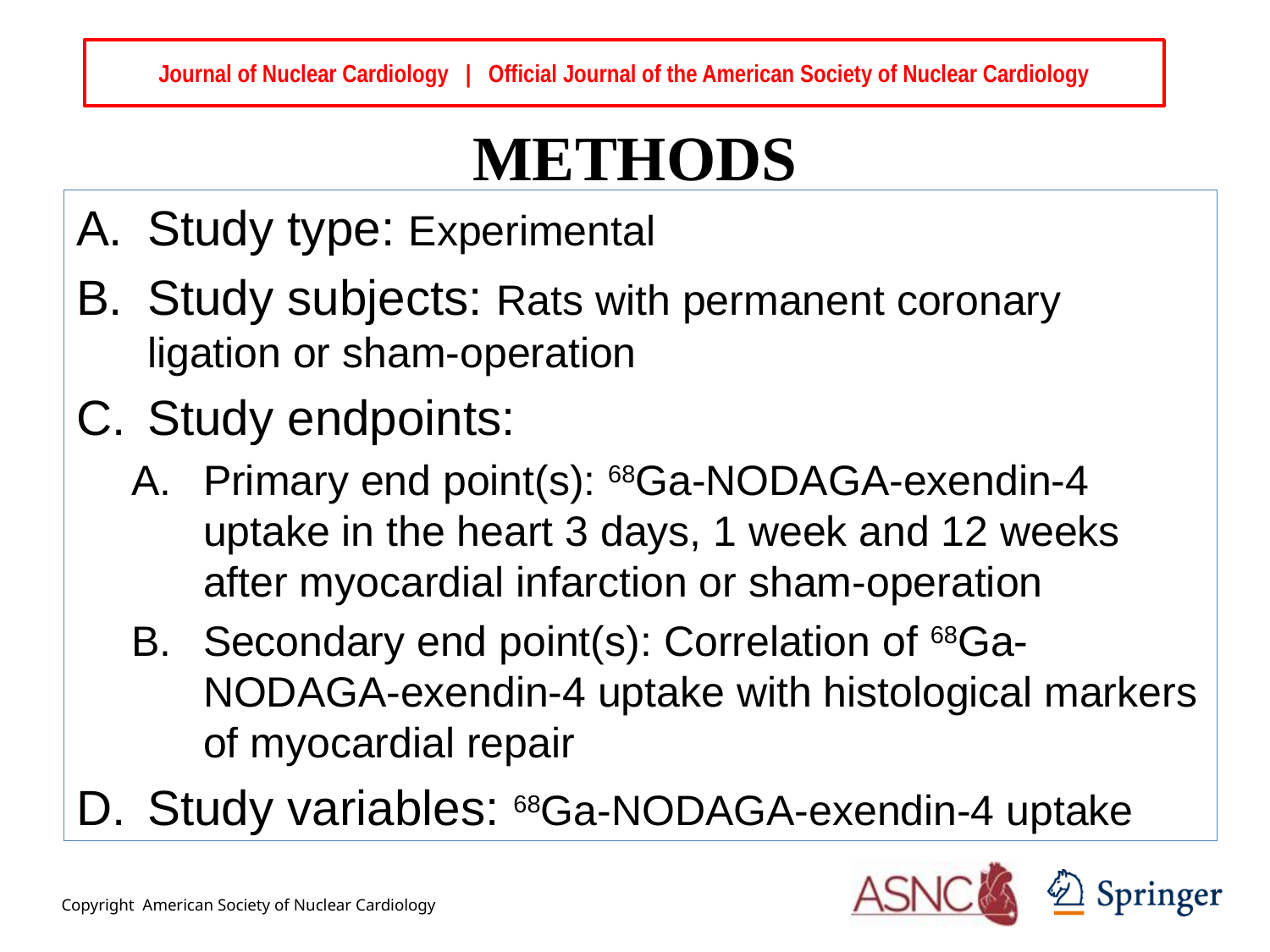

Journal of Nuclear Cardiology | Official Journal of the American Society of Nuclear Cardiology
# METHODS
Study type: Experimental
Study subjects: Rats with permanent coronary ligation or sham-operation
Study endpoints:
Primary end point(s): 68Ga-NODAGA-exendin-4 uptake in the heart 3 days, 1 week and 12 weeks after myocardial infarction or sham-operation
Secondary end point(s): Correlation of 68Ga-NODAGA-exendin-4 uptake with histological markers of myocardial repair
Study variables: 68Ga-NODAGA-exendin-4 uptake
Copyright American Society of Nuclear Cardiology

## Slide 4
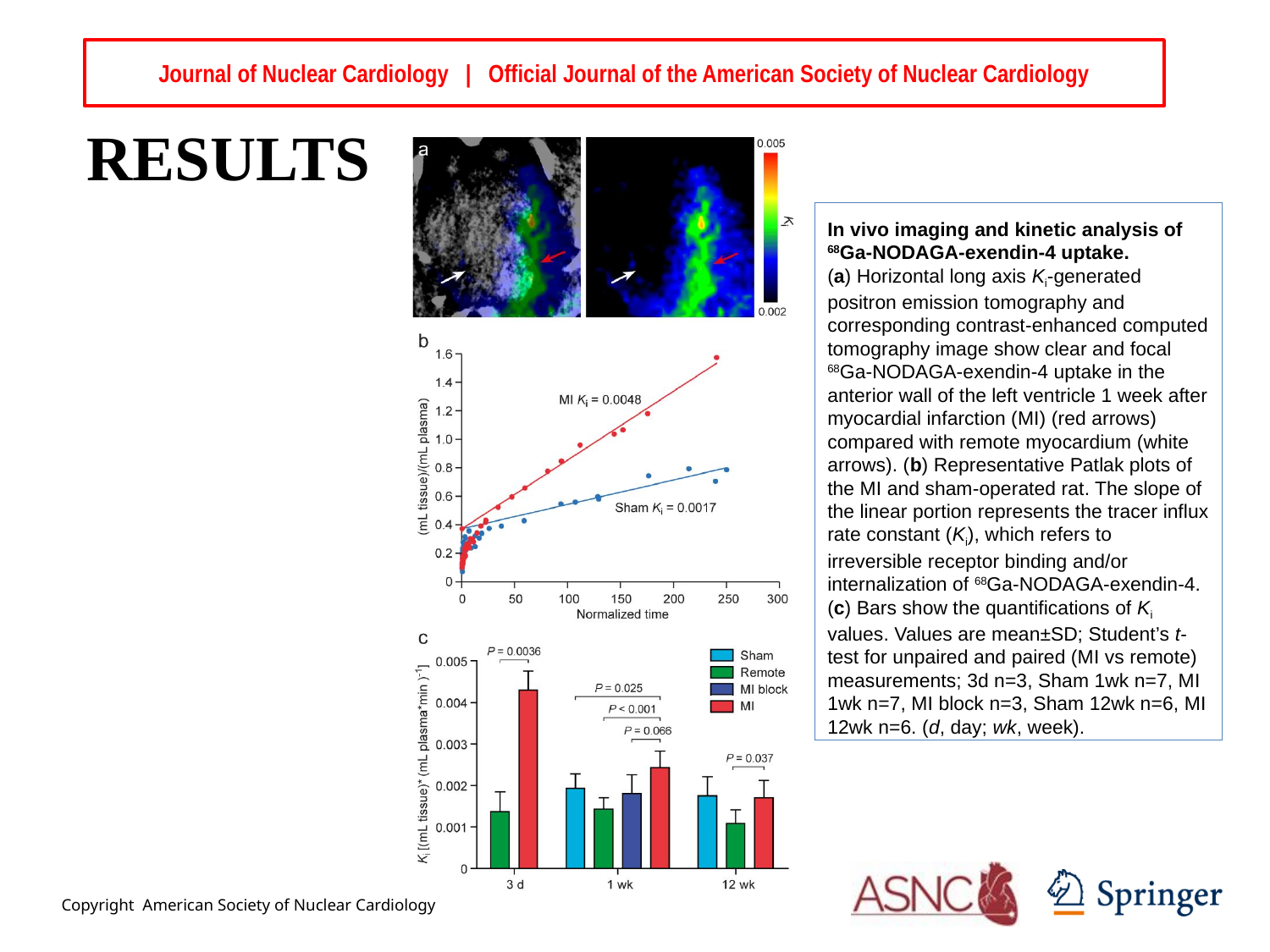

Journal of Nuclear Cardiology | Official Journal of the American Society of Nuclear Cardiology
# RESULTS
In vivo imaging and kinetic analysis of 68Ga-NODAGA-exendin-4 uptake.
(a) Horizontal long axis Ki-generated positron emission tomography and corresponding contrast-enhanced computed tomography image show clear and focal 68Ga-NODAGA-exendin-4 uptake in the anterior wall of the left ventricle 1 week after myocardial infarction (MI) (red arrows) compared with remote myocardium (white arrows). (b) Representative Patlak plots of the MI and sham-operated rat. The slope of the linear portion represents the tracer influx rate constant (Ki), which refers to irreversible receptor binding and/or internalization of 68Ga-NODAGA-exendin-4. (c) Bars show the quantifications of Ki values. Values are mean±SD; Student’s t-test for unpaired and paired (MI vs remote) measurements; 3d n=3, Sham 1wk n=7, MI 1wk n=7, MI block n=3, Sham 12wk n=6, MI 12wk n=6. (d, day; wk, week).
Copyright American Society of Nuclear Cardiology

## Slide 5
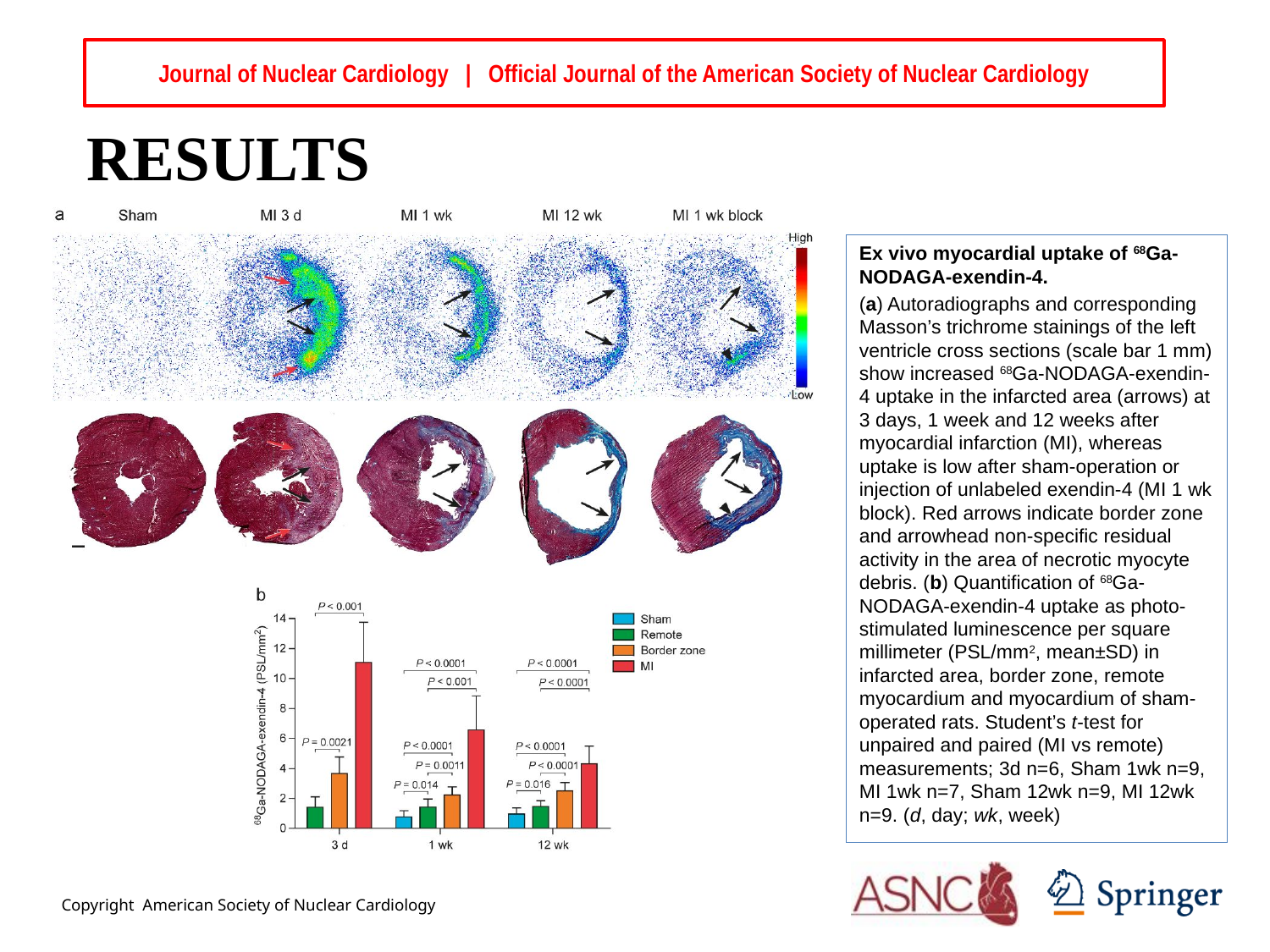

Journal of Nuclear Cardiology | Official Journal of the American Society of Nuclear Cardiology
# RESULTS
Ex vivo myocardial uptake of 68Ga-NODAGA-exendin-4.
(a) Autoradiographs and corresponding Masson’s trichrome stainings of the left ventricle cross sections (scale bar 1 mm) show increased 68Ga-NODAGA-exendin-4 uptake in the infarcted area (arrows) at 3 days, 1 week and 12 weeks after myocardial infarction (MI), whereas uptake is low after sham-operation or injection of unlabeled exendin-4 (MI 1 wk block). Red arrows indicate border zone and arrowhead non-specific residual activity in the area of necrotic myocyte debris. (b) Quantification of 68Ga-NODAGA-exendin-4 uptake as photo-stimulated luminescence per square millimeter (PSL/mm2, mean±SD) in infarcted area, border zone, remote myocardium and myocardium of sham-operated rats. Student’s t-test for unpaired and paired (MI vs remote) measurements; 3d n=6, Sham 1wk n=9, MI 1wk n=7, Sham 12wk n=9, MI 12wk n=9. (d, day; wk, week)
Copyright American Society of Nuclear Cardiology

## Slide 6
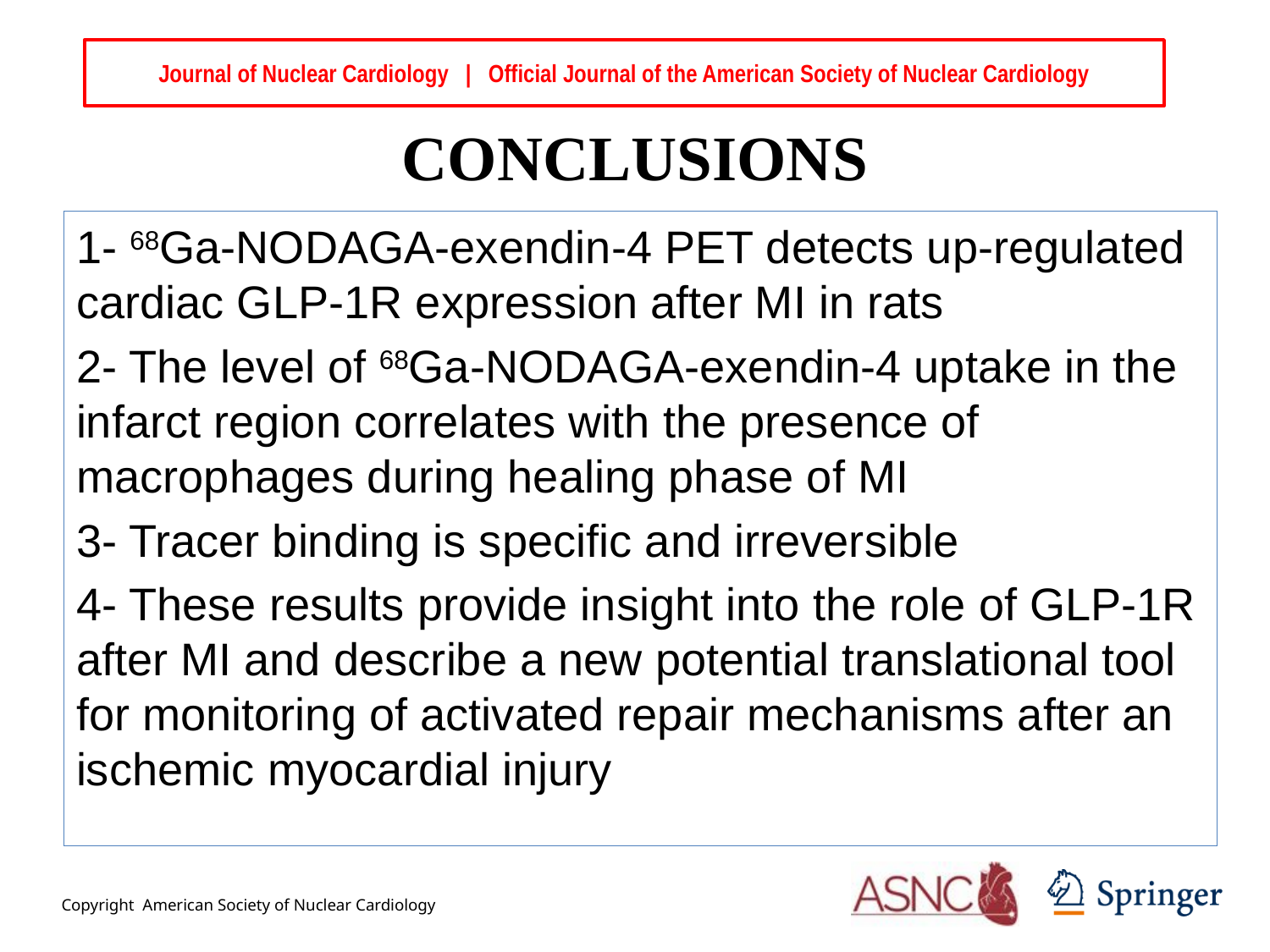

Journal of Nuclear Cardiology | Official Journal of the American Society of Nuclear Cardiology
# CONCLUSIONS
1- 68Ga-NODAGA-exendin-4 PET detects up-regulated cardiac GLP-1R expression after MI in rats
2- The level of 68Ga-NODAGA-exendin-4 uptake in the infarct region correlates with the presence of macrophages during healing phase of MI
3- Tracer binding is specific and irreversible
4- These results provide insight into the role of GLP-1R after MI and describe a new potential translational tool for monitoring of activated repair mechanisms after an ischemic myocardial injury
Copyright American Society of Nuclear Cardiology
